# Clinical and neuroimaging correlates of motoric cognitive risk syndrome in cerebral small vessel disease: a cross-sectional study

**DOI:** 10.3389/fnagi.2026.1853965

**Published:** 2026-06-11

**Authors:** Huixin Zhang, Tong Guo, Ran Zhang, Hongyi Yan, Shuo Yang, Ying Gao, Tingting Wang, Weiqi Chen, Yuesong Pan, Yilong Wang, Weixin Cai, Ling Guan

**Affiliations:** 1 Department of Nursing, Beijing Tiantan Hospital, Beijing, China; 2 Department of Neurology, Beijing Tiantan Hospital, Beijing, China; 3 National Clinical Research Center for Neurological Diseases, Beijing Tiantan Hospital, Beijing, China; 4 School of Interdisciplinary Science, Beijing Institute of Technology, Beijing, China; 5 Department of Medicine, The University of British Columbia, Vancouver, BC, Canada

**Keywords:** cerebral small vessel disease, motoric cognitive risk syndrome, neuroimaging, physical activity, slow gait, subjective cognitive complaints

## Abstract

**Introduction:**

Cerebral small vessel disease (CSVD) and motoric cognitive risk syndrome (MCR) are both associated with adverse outcomes in older adults. Factors associated with MCR in CSVD remain poorly understood. We aimed to explore factors associated with MCR and its components (slow gait and subjective cognitive complaints [SCCs]) in CSVD, and to evaluate exploratory association-based multivariable models.

**Methods:**

This cross-sectional study included CSVD patients aged ≥55 years without possible dementia based on education-adjusted MoCA screening from the cognitive subgroup of a national registry. Demographics, clinical variables, physical activity, and CSVD neuroimaging markers were assessed. MCR was defined as co-existing slow gait (age- and sex-adjusted) and SCCs (single-item memory complaint from the 15-item Geriatric Depression Scale). Logistic regression and receiver operating characteristic analyses were performed to identify associated factors and evaluate models. Firth penalized logistic regression and bootstrap internal validation were additionally performed to assess sparse-data bias and optimism in model discrimination.

**Results:**

Among 225 patients (mean age 65.4 years, 54.2% male), MCR prevalence was 16.4%. In multivariable models, MCR was associated with lower average systolic blood pressure and high total CSVD burden, while physical inactivity showed a positive but imprecise association because of sparse exposure. SCCs were associated with greater juxtacortical white matter hyperintensity volume and higher total CSVD burden. Slow gait was associated with dyslipidemia, poorer functional status, and higher basal ganglia perivascular spaces. The comprehensive exploratory model integrating demographic, clinical, and neuroimaging factors achieved areas under the curve of 0.732 for MCR, 0.733 for SCCs, and 0.770 for slow gait; the corresponding optimism-corrected AUCs were 0.691, 0.696, and 0.725, respectively.

**Conclusion:**

In this exploratory cross-sectional analysis, MCR in CSVD patients showed associations with vascular, lifestyle-related, and neuroimaging markers. However, given the small number of MCR events, the single-item SCC assessment, the lack of temporality, and the limited persistence of associations after FDR correction, these findings should be interpreted as hypothesis-generating.

## 1 Introduction

Cerebral small vessel disease (CSVD) is a common cerebrovascular disorder that contributes to vascular cognitive impairment and dementia (VCID) and gait disturbance in older adults (Sharma et al., 2023; Sachdev et al., 2025; Saks and Sachdev, 2025). Structural magnetic resonance imaging (MRI) is the gold-standard tool for identifying CSVD neuroimaging features clinically (Duering et al., 2023). Although the association between VCID and Alzheimer’s disease (AD) remains debated, evidence suggests that CSVD-related imaging markers may increase the risk of AD in older adults and interact with AD pathology (Kim et al., 2022; Twait et al., 2023).

Motoric cognitive risk syndrome (MCR) is a pre-dementia state characterized by the coexistence of slow gait and subjective cognitive complaints (SCCs) in individuals without dementia (Verghese et al., 2014; Verghese et al., 2019). It represents a transitional stage between normal ageing, mild cognitive impairment and dementia (Verghese et al., 2013). The global prevalence of MCR in the older population is approximately 9.7%, with estimates ranging from 2 to 18% across populations (Lim et al., 2024). MCR has been associated with adverse outcomes such as dementia, falls, and death (Verghese et al., 2013; Bai et al., 2022; Huang et al., 2024; Lim et al., 2024). Early detection of MCR may help delay cognitive decline and reduce dementia risk (Lim et al., 2024).

CSVD and MCR share overlapping clinical features and pathological mechanisms. White matter damage and cerebral hypoperfusion in CSVD may impair gait-regulatory networks and cognitive-related brain regions, contributing to both motor and cognitive decline (Sathyan et al., 2023; Hagiwara et al., 2025). However, most studies on MCR have focused on the general population. Evidence on MCR in CSVD remains relatively scarce, and the clinical and neuroimaging correlates of MCR in this group are not well understood. The correlates of MCR in CSVD patients are likely multifactorial (Zhang et al., 2020; Azevedo et al., 2025), including traditional vascular risk factors, CSVD neuroimaging markers, as well as lifestyle factors such as physical activity.

Currently, the diagnosis of MCR mainly relies on gait speed measurement and SCC assessment (Xiang et al., 2021). However, in a CSVD population, where patients may have varying degrees of physical disability (e.g., from prior strokes or general frailty), completing a standardized gait test may not always be feasible. This creates a need for alternative approaches to identifying patients who may warrant closer cognitive-motor assessment even when formal gait testing is unavailable. The study aimed to: 1) examine exploratory factors associated with MCR and its individual components (slow gait and SCCs) in CSVD patients; and 2) construct and internally evaluate exploratory association-based multivariable models to characterize associated clinical, lifestyle-related, and neuroimaging profiles among CSVD patients.

## 2 Materials and methods

### 2.1 Study design and participants

This was a cross-sectional study using data from the cognitive subgroup of a national CSVD registry in China. The present analysis was restricted to participants with complete assessments of SCCs, gait, cognition, and baseline neuroimaging data, which were available from the Beijing Tiantan Hospital cognitive subgroup. Patients with CSVD neuroimaging manifestations were enrolled from January 2020 to March 2022 and scheduled for annual follow-up for five consecutive years. The detailed inclusion and exclusion criteria have been published elsewhere (Zhao et al., 2024). Briefly, we recruited patients aged >18 years who had WMHs of presumed vascular origin with a Fazekas score ≥2, or a Fazekas score of 1 combined with at least two vascular risk factors (i.e., hypertension, dyslipidemia, diabetes, obesity, or smoking) or subcortical lacunar infarctions. All participants underwent baseline clinical assessment by trained neurologists. Patients were excluded if they had functional dependency, defined as a modified Rankin Scale (mRS) score >2, recent cerebral infarction >20 mm in diameter confirmed by diffusion-weighted imaging, acute intracerebral hemorrhage or subarachnoid hemorrhage, a previous clinical diagnosis of Parkinson’s disease, parkinsonism, other neurodegenerative diseases, or WMHs of non-vascular origin.

This study was approved by the Institutional Review Board of Beijing Tiantan Hospital (Approval Number: KY201914002; Approval Date: December 29, 2019) and conducted in accordance with the World Medical Association Declaration of Helsinki. All patients or their legally authorized representatives provided written informed consent prior to enrolment. The study was registered with the Chinese Clinical Trial Registry (ChiCTR2100043346).

### 2.2 Study assessment

#### 2.2.1 Assessment of demographic and clinical information

At baseline, we collected demographic and basic clinical information from the enrolled patients. Demographics included age, sex, years of education, marital status, living situation (solitude), insurance status and average income level. Average income level was categorized as low (monthly household income per capita <2000 CNY), moderate (2000 to <6,000 CNY), and high (≥6,000 CNY). Vital signs (including body mass index [BMI] and blood pressure [systolic blood pressure [SBP] and diastolic blood pressure [DBP]), past medical history, cigarette and alcohol consumption, and functional independence [assessed by the mRS score (Quinn et al., 2009)] were also collected. Specifically, we focused on physical-activity-related variables, including participation in any form or at specific levels of physical activity, as well as the average intensity. According to the 2020 WHO guidelines on physical activity and sedentary behavior (Ainsworth et al., 2011; WHO, 2020), physical activity intensity was defined as light (1.5 to <3 metabolic equivalents of task [METs]), moderate (3 to 6 METs), and vigorous (≥6 METs).

#### 2.2.2 Assessment of CSVD neuroimaging features

Patients underwent brain MRI examination using a 3.0-T scanner (Philips, The Netherlands) at Beijing Tiantan Hospital or their respective centers. Standard sequences included axial T1-weighted, T2-weighted, fluid-attenuated inversion recovery (FLAIR), diffusion-weighted imaging (DWI), MR angiography, and susceptibility-weighted imaging (SWI) or gradient-recalled echo sequences. Two vascular neurologists independently evaluated all images for CSVD-related markers, including WMHs, lacunes, cerebral microbleeds (CMBs), enlarged perivascular spaces (ePVS), and recent small subcortical infarcts (RSSIs). Standardized quantitative volumetric measures or harmonized visual ratings of brain atrophy were not available in the final analytical dataset; therefore, brain atrophy was not included in the present analysis. Discrepancies were resolved by consensus discussion with a third neurologist.

WMH severity was rated semi-quantitatively using the MRI-based Fazekas score (Fazekas et al., 1987) by two trained neurologists. The total Fazekas score ranges from 0 to 6 and is the sum of the periventricular and deep WMH scores. Periventricular WMHs were graded as 0 if absent, 1 for “caps” or pencil-thin lining, 2 for smooth “halo,” and 3 for irregular WMHs extending into the deep white matter. Deep WMHs were graded as 0 if absent, 1 for punctate foci, 2 when foci begin to confluence, and 3 for large confluent areas. The total Fazekas scores were divided into three categories: 1–2, 3–4, and 5–6. Higher Fazekas scores or grades indicate greater WMH severity. Quantitatively, the absolute volume of WMH (in milliliters) and its percentage relative to the total cerebral white matter volume were calculated. These measurements were obtained using the fully automated UBO Detector toolbox. Briefly, after co-registration of T2-weighted to T1-weighted images and tissue segmentation, images were normalized to a study-specific template using the Diffeomorphic Anatomical Registration Through Exponentiated Lie (DARTEL) algorithm. WMHs were then automatically identified in the normalized space using a k-nearest neighbor (k-NN) machine learning algorithm. All automated processing steps underwent meticulous quality control through review of the generated HTML reports by an experienced neurologist.

CSVD markers were defined according to the updated STRIVE-2 neuroimaging standards (Duering et al., 2023). Lacunes were defined as round or ovoid, subcortical, fluid-filled cavities, typically 3–15 mm in diameter, with signal intensity similar to cerebrospinal fluid on T1-weighted, T2-weighted, and FLAIR images, usually with a surrounding hyperintense rim on FLAIR. CMBs were defined as small, rounded or ovoid hypointense lesions on SWI or gradient-recalled echo sequences, generally ≤10 mm in diameter, after excluding mimics such as vascular flow voids, calcification, and imaging artifacts. ePVS were defined as small, sharply demarcated, punctate or linear structures with cerebrospinal-fluid-like signal intensity along the course of perforating vessels, and were counted in predefined anatomical regions, including the basal ganglia and centrum semiovale. RSSIs were defined as recent small subcortical infarcts in the territory of one perforating arteriole, identified mainly on DWI according to lesion size and location.

The total CSVD burden score (TB-CSVD) was calculated according to Staals et al. (2014), based on four markers: severe WMHs, CMBs, ePVS score in the basal ganglia (BG-ePVS), and lacunes. The TB-CSVD ranges from 0 to 4, with one point assigned for each of the following criteria: (1) Fazekas score for periventricular WMHs ≥3 or deep WMHs ≥2, (2) CMB count ≥1, (3) BG-ePVS count >10, (4) lacune count ≥1. Higher TB-CSVD scores indicate a greater neuroimaging burden of CSVD. For analysis, the TB-CSVD was dichotomized into low (scores 0–1) and high (scores 2–4) neuroimaging burden groups.

#### 2.2.3 Assessment of motoric cognitive risk syndrome

MCR was defined as the coexistence of SCCs and slow gait in individuals aged ≥55 years without possible dementia or mobility disability (Verghese et al., 2013). In this study, SCCs referred specifically to single-item memory complaints used for pragmatic MCR operationalization. SCCs were evaluated through face-to-face interviews using a single memory-related item from the 15-item Geriatric Depression Scale (GDS-15) (Yesavage et al., 1982): “Do you feel you have more problems with memory than most?” A “yes” response indicated the presence of SCCs. This single-item memory complaint question has been used in previous MCR studies, including a CSVD-focused MCR study and studies in older Chinese and other population-based cohorts (Wang et al., 2016; Lau et al., 2019; Shen et al., 2020; Zeng et al., 2021), and was feasible within the registry-based assessment. However, it should be regarded as a pragmatic SCC measure rather than a psychometrically comprehensive multidimensional assessment of subjective cognitive decline (Jessen et al., 2014; Jessen et al., 2020). Gait speed was assessed using a 6-meter walk test (Studenski et al., 2011). Participants walked a 6-meter course at their usual, comfortable speed. The time to cover the middle 4 meters was used to calculate gait speed meters per second (m/s). Slow gait was defined as gait speed more than one standard deviation below age- and sex-specific mean values. The cut-off values for slow gait in this study (Tian et al., 2016) were: for males aged 55–64 years, 0.87 m/s; 65–74 years, 0.79 m/s; 75–85 years, 0.70 m/s; for females, the values were 0.86 m/s, 0.75 m/s, and 0.68 m/s for the same age groups, respectively. Possible dementia was screened using the full version of the Montreal Cognitive Assessment (MoCA-full) scale (Nasreddine et al., 2005). An education-adjusted MoCA-full score below 18 was used pragmatically to indicate possible dementia, and participants below this threshold were excluded from this analysis. The total MoCA-full score was adjusted for education by adding 1 point for participants with fewer than 12 years of formal education.

Based on these assessments, participants were first categorized dichotomously (with or without MCR, SCCs, or slow gait). They were then classified into four mutually exclusive groups according to their gait speed and SCC status: Group A (MCR), slow gait with SCCs; Group B, normal gait with SCCs; Group C, slow gait without SCCs; and Group D, normal gait without SCCs.

### 2.3 Statistical analysis

Demographic, clinical and CSVD neuroimaging characteristics were summarised as categorical or continuous variables, and were compared between groups with versus without MCR (Group A vs. Group B + C + D), SCCs (Group A + B vs. Group C + D), and slow gait (Group A + C vs. Group B + D). Absolute volumes of WMHs, CMBs, and RSSIs were rescaled and entered into logistic regression models per 1,000 mm^3^ to improve interpretability of ORs. For logistic regression analyses, binary outcomes were coded as 1 for the presence and 0 for the absence of the outcome; odds ratios therefore indicate the odds of MCR, SCCs, or slow gait. Candidate variables for exploratory association-based multivariable models were selected based on a combination of clinical relevance, biological plausibility, and statistical evidence from univariable analyses, rather than on univariable *p* values alone. Age, sex, and years of education were retained *a priori* as basic demographic covariates in all models. Receiver operating characteristic (ROC) curves and the corresponding area under the curve (AUC) were used to evaluate model discrimination. Pairwise comparisons of AUCs between models were performed using DeLong tests. Given the large number of univariable comparisons, false discovery rate (FDR) adjustment using the Benjamini-Hochberg method was additionally performed for multiplicity-aware interpretation of univariable screening results. We additionally performed Firth penalized logistic regression for the multivariable models to reduce small-sample and sparse-data bias. Bootstrap internal validation with 1,000 resamples was performed to estimate optimism-corrected AUCs. Sensitivity analyses were also conducted using alternative physical activity metrics, including no moderate-to-vigorous physical activity, moderate-to-vigorous physical activity, and a four-level physical-activity intensity variable. Missing data were not imputed. Analyses were performed on complete cases only, as the proportion of missing data was low (<5% for all variables). The final analytical sample comprised 225 participants with complete data for all variables of interest. A two-sided *p*-value <0.05 was considered statistically significant, whereas FDR-adjusted results and DeLong tests were interpreted as supplementary exploratory evidence. All statistical analyses were performed using R software, version 4.3.2.

## 3 Results

### 3.1 Baseline characteristics of participants

**Figure 1:**
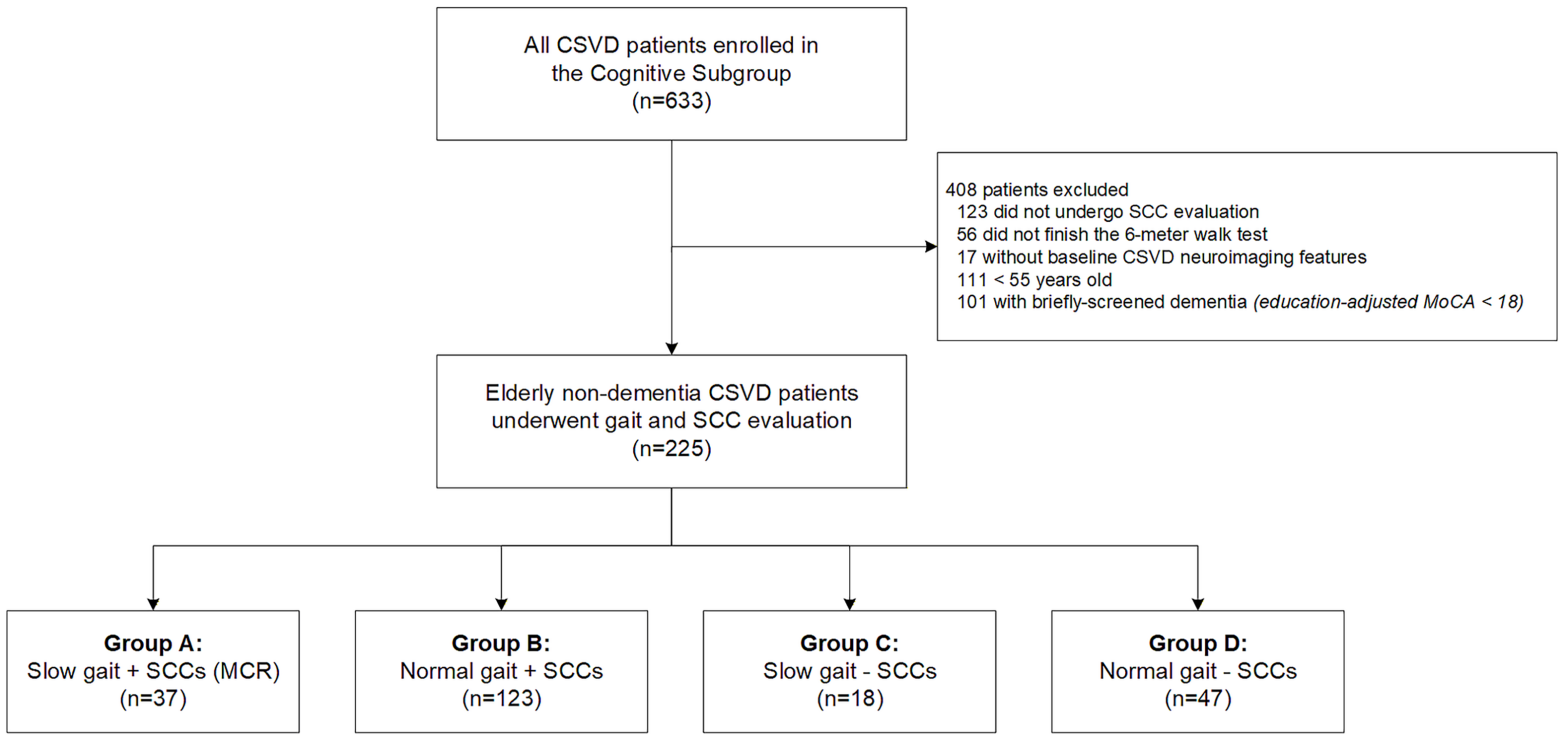
Flowchart. CSVD, cerebral small vessel disease; MCR, motoric cognitive risk syndrome; MoCA, the Montreal Cognitive Assessment Scale; SCCs, subjective cognitive complaints.

A total of 633 patients with CSVD from the cognitive sub-cohort were identified for potential inclusion. Among them, 123 patients did not undergo SCC evaluation, and a further 56 patients did not complete the 6-meter walk test. An additional 17 patients were excluded due to unavailable baseline CSVD neuroimaging data, 111 were aged <55 years, and 101 were identified as possible dementia based on education-adjusted MoCA scores. Consequently, 225 CSVD patients aged ≥55 years who had completed both gait and SCC assessments and did not meet the MoCA-based screening criterion for possible dementia were included in the final analysis (Figure 1). Among these, 37 patients were categorized into Group A, defined by the presence of both slow gait and SCCs (MCR). No missing data were present for the variables included in the multivariable models after application of the exclusion criteria.

### 3.2 Comparisons of demographic, clinical and neuroimaging-based parameters among groups

Demographic, clinical, and neuroimaging characteristics stratified by MCR, SCCs, and slow-gait status are summarized in Tables 1, 2. In the demographic and clinical comparisons, several differences were observed across the four mutually exclusive groups and in the corresponding binary comparisons. Patients with MCR had lower average SBP, lower participation in any physical activity, worse functional status as indicated by mRS score, and a higher prevalence of ICH than those without MCR. Patients with SCCs differed from those without SCCs in marital status, DBP, diabetes, and activity-intensity distribution. Patients with slow gait had lower SBP and DBP, lower participation in moderate- or vigorous-intensity physical activity, and worse functional status than those without slow gait.

**Table 1:** Demographic and clinical information of included CSVD patients with or without MCR, SCCs, and slow gait.

Variable	Level	Total (*n =* 225)	Group A (MCR): Slow gait + SCCs (*n =* 37)	Group B: Normal gait + SCCs (*n =* 123)	Group C: Slow gait – SCCs (*n =* 18)	Group D: Normal gait – SCCs (*n =* 47)	Overall *P*	P^*^	*P* ^⁑^	*P* ^⁂^
Demographics										
Age, years		65.00 (59.00, 71.00)	64.00 (58.00, 68.00)	65.00 (59.00, 69.50)	66.50 (61.25, 72.75)	67.00 (61.50, 71.50)	0.067	0.110	**0.012**	0.483
Female, *n* (%)		85 (37.8)	16 (43.2)	45 (36.6)	9 (50.0)	15 (31.9)	0.493	0.464	0.881	0.202
Years of education, M (Q₁, Q₃)		12.00 (9.00, 12.00)	12.00 (9.00, 12.00)	12.00 (9.00, 12.00)	12.00 (9.50, 12.00)	12.00 (9.00, 15.00)	0.149	0.146	**0.035**	0.424
Married, *n* (%)		211 (93.8)	37 (100.0)	117 (95.1)	15 (83.3)	42 (89.4)	**0.035**	0.134	**0.029**	1.000
Solitary, *n* (%)		13 (5.8)	1 (2.7)	7 (5.7)	2 (11.1)	3 (6.4)	0.588	0.699	0.529	1.000
Insured, *n* (%)		217 (96.4)	36 (97.3)	119 (96.7)	18 (100.0)	44 (93.6)	0.703	1.000	0.693	0.683
Average income level ^a^, *n* (%)	Low	27 (12.0)	8 (21.6)	12 (9.8)	3 (16.7)	4 (8.5)	0.319	0.087	0.771	**0.047**
	Moderate	172 (76.4)	27 (73.0)	96 (78.0)	14 (77.8)	35 (74.5)				
	High	26 (11.6)	2 (5.4)	15 (12.2)	1 (5.6)	8 (17.0)				
Vital signs										
BMI, Mean ±SD		24.65 ± 2.92	25.04 ± 2.75	24.63 ± 2.92	24.69 ± 3.11	24.36 ± 3.04	0.770	0.374	0.518	0.417
Right SBP, M (Q₁, Q₃)		134.00 (125.00, 145.00)	130.00 (125.00, 139.00)	138.00 (125.00, 147.00)	130.00 (123.50, 134.25)	137.00 (126.00, 149.50)	0.139	0.100	0.870	**0.024**
Right DBP, M (Q₁, Q₃)		82.00 (75.00, 89.00)	80.00 (76.00, 89.00)	85.00 (78.00, 91.50)	78.00 (71.00, 84.00)	80.00 (73.50, 88.50)	**0.028**	0.700	**0.013**	0.075
Left SBP, M (Q₁, Q₃)		133.00 (123.00, 145.00)	128.00 (120.00, 133.00)	134.00 (124.50, 148.00)	128.50 (122.25, 137.25)	135.00 (125.50, 150.00)	**0.014**	**0.004**	0.607	**0.001**
Left DBP, M (Q₁, Q₃)		82.00 (75.00, 89.00)	82.00 (74.00, 88.00)	85.00 (78.00, 90.00)	74.00 (71.25, 81.50)	79.00 (72.50, 88.00)	**0.007**	0.482	**0.005**	**0.028**
Average SBP, M (Q₁, Q₃)		132.50 (124.00, 144.00)	128.00 (122.50, 135.00)	135.50 (125.00, 146.50)	129.00 (124.12, 135.50)	135.00 (126.25, 149.50)	**0.032**	**0.015**	0.846	**0.003**
Average DBP, M (Q₁, Q₃)		82.50 (75.50, 88.50)	81.50 (76.00, 86.00)	84.00 (77.75, 90.75)	76.75 (71.50, 83.38)	79.00 (73.50, 87.00)	**0.010**	0.579	**0.010**	**0.040**
Past medical history										
Current smoking, *n* (%)		42 (18.7)	4 (10.8)	25 (20.3)	1 (5.6)	12 (25.5)	0.175	0.249	0.850	**0.045**
Current alcohol consumption, *n* (%)		32 (14.2)	3 (8.1)	14 (11.4)	1 (5.6)	14 (29.8)	**0.012**	0.310	**0.020**	0.119
Diabetes, *n* (%)		63 (28.0)	8 (21.6)	26 (21.1)	8 (44.4)	21 (44.7)	**0.006**	0.425	**0.001**	0.864
Hypertension, *n* (%)		162 (72.0)	30 (81.1)	86 (69.9)	11 (61.1)	35 (74.5)	0.399	0.230	0.870	0.731
Dyslipidemia, *n* (%)		64 (28.4)	17 (45.9)	31 (25.2)	5 (27.8)	11 (23.4)	0.091	**0.016**	0.515	**0.039**
All stroke, *n* (%)		92 (40.9)	19 (51.4)	50 (40.7)	6 (33.3)	17 (36.2)	0.484	0.200	0.299	0.435
Ischemic stroke, *n* (%)		88 (39.1)	16 (43.2)	50 (40.7)	5 (27.8)	17 (36.2)	0.701	0.585	0.366	1.000
ICH, *n* (%)		4 (1.8)	3 (8.1)	0 (0.0)	1 (5.6)	0 (0.0)	**0.005**	**0.015**	1.000	**0.003**
TIA, *n* (%)		12 (5.3)	1 (2.7)	8 (6.5)	0 (0.0)	3 (6.4)	0.793	0.696	1.000	0.302
Coronary heart disease, *n* (%)		31 (13.8)	2 (5.4)	19 (15.4)	2 (11.1)	8 (17.0)	0.400	0.124	0.672	0.121
Atrial fibrillation, *n* (%)		4 (1.8)	0 (0.0)	3 (2.4)	0 (0.0)	1 (2.1)	1.000	1.000	1.000	0.574
Headache, *n* (%)		38 (16.9)	6 (16.2)	27 (22.0)	1 (5.6)	4 (8.5)	0.111	1.000	**0.019**	0.412
Physical activity										
Any physical activity, *n* (%)		218 (96.9)	33 (89.2)	121 (98.4)	18 (100.0)	46 (97.9)	0.058	**0.015**	0.676	0.063
Average intensity ^b^, *n* (%)	Vigorous	12 (5.3)	5 (13.51)	5 (4.07)	1 (5.56)	1 (2.13)	**0.018**	**0.042**	**0.027**	**0.023**
	Moderate	132 (58.7)	18 (48.65)	83 (67.48)	7 (38.89)	24 (51.06)				
	Light	81 (36.0)	14 (37.84)	35 (28.46)	10 (55.56)	22 (46.81)				
Vigorous-intensity physical activity, *n* (%)		63 (28.0)	6 (16.2)	40 (32.5)	2 (11.1)	15 (31.9)	0.085	0.108	0.745	**0.010**
Moderate-intensity physical activity, *n* (%)		147 (65.3)	20 (54.1)	87 (70.7)	7 (38.9)	33 (70.2)	**0.024**	0.132	0.445	**0.005**
Light-intensity physical activity, *n* (%)		125 (55.6)	17 (45.9)	68 (55.3)	11 (61.1)	29 (61.7)	0.521	0.210	0.301	0.439
Functional independence										
mRS, *n* (%)	0	144 (64.0)	16 (43.2)	83 (67.5)	10 (55.6)	35 (74.5)	**0.029**	**0.011**	0.388	**0.006**
	1	73 (32.4)	19 (51.4)	37 (30.1)	6 (33.3)	11 (23.4)				
	2	8 (3.6)	2 (5.4)	3 (2.4)	2 (11.1)	1 (2.1)				

^a^Average income level was defined as: Low (monthly household income per capita <2000 CNY), Moderate (monthly household income per capita 2000- < 6,000 CNY), and High (monthly household income per capita ≥6,000 CNY). ^b^According to WHO guidelines on physical activity and sedentary behavior in 2020, physical activity intensity was defined as: Light-intensity (1.5–<3 metabolic equivalent of tasks [METs]), Moderate-intensity (3–6 METs), and Vigorous-intensity (≥6 METs). Overall P: P for the comparison among the four groups, P^*^: P for the comparison between CSVD with or without MCR (Group A vs Group B + C + D), P^⁑^: P for the comparison between CSVD with or without SCCs (Group A + B vs Group C + D), and P^⁂^: P for the comparison between CSVD with or without slow gait (Group A + C vs Group B + D). Bold values indicate *P* < 0.05. BMI, body mass index; CSVD, cerebral small vessel disease; DBP, diastolic blood pressure; ICH, intracerebral hemorrhage; MCR, motoric cognitive risk syndrome; M, Median; mRS, modified Rankin Scale; n, number; Q₁, 1st Quartile; Q₃, 3rd Quartile; SBP, systolic blood pressure; SCCs, subjective cognitive complaints; SD, standard deviation; TIA: transient ischemic attack.

**Table 2:** CSVD neuroimaging parameters of included CSVD patients with or without MCR, SCCs, and slow gait (*P* < 0.05).

Variable	Level	Total (*n =* 225)	Group A (MCR): Slow gait + SCCs (*n =* 37)	Group B: Normal gait + SCCs (*n =* 123)	Group C: Slow gait – SCCs (*n =* 18)	Group D: Normal gait – SCCs (*n =* 47)	Overall *P*	P^*^	*P^⁑^*	*P^⁂^*
Individual neuroimaging markers										
Total ePVS count, M (Q₁, Q₃)		69.00 (34.00, 104.00)	78.00 (38.00, 100.00)	59.00 (26.50, 100.50)	90.00 (69.50, 123.75)	70.00 (40.00, 103.50)	**0.040**	0.551	**0.035**	**0.048**
Right frontal lobe PVS count, M (Q₁, Q₃)		28.00 (15.00, 48.00)	30.00 (16.00, 49.00)	25.00 (11.50, 45.00)	42.50 (23.50, 53.25)	32.00 (21.00, 48.50)	0.114	0.874	**0.043**	0.174
Left parietal lobe PVS count, M (Q₁, Q₃)		19.00 (8.00, 32.00)	19.00 (12.00, 32.00)	16.00 (5.00, 31.00)	30.50 (17.50, 35.75)	21.00 (8.50, 30.00)	**0.026**	0.511	**0.041**	**0.028**
Right parietal lobe PVS count, M (Q₁, Q₃)		22.00 (9.00, 32.00)	18.00 (9.00, 29.00)	20.00 (5.00, 32.00)	32.00 (24.50, 42.00)	20.00 (9.50, 29.50)	**0.012**	0.629	0.057	0.099
Left BG PVS count, M (Q₁, Q₃)		21.00 (13.00, 29.00)	25.00 (18.00, 31.00)	21.00 (13.00, 27.00)	19.50 (13.50, 25.25)	18.00 (13.00, 27.00)	0.130	**0.020**	0.280	0.083
Right BG PVS count, M (Q₁, Q₃)		19.00 (13.00, 28.00)	24.00 (15.00, 30.00)	18.00 (13.00, 27.00)	18.00 (12.25, 23.00)	19.00 (12.50, 26.50)	0.202	**0.032**	0.483	0.124
Left CSO PVS count, M (Q₁, Q₃)		12.00 (5.00, 22.00)	13.00 (6.00, 27.00)	11.00 (3.00, 21.00)	17.50 (11.75, 26.75)	11.00 (6.00, 17.00)	0.087	0.390	0.356	**0.029**
Overall parietal lobe PVS count, M (Q₁, Q₃)		40.00 (20.00, 63.00)	42.00 (22.00, 58.00)	37.00 (12.50, 61.50)	61.00 (42.25, 74.50)	38.00 (20.50, 59.50)	**0.019**	0.958	**0.046**	0.052
Overall BG PVS count, M (Q₁, Q₃)		40.00 (29.00, 57.00)	49.00 (35.00, 65.00)	40.00 (28.50, 53.00)	34.50 (29.25, 49.25)	35.00 (24.00, 51.50)	0.128	**0.019**	0.280	0.101
Lacune count (based on FLAIR), M (Q₁, Q₃)		1.00 (0.00, 3.00)	2.00 (0.00, 6.00)	1.00 (0.00, 3.00)	1.00 (0.00, 2.00)	1.00 (0.00, 2.00)	0.160	**0.046**	0.109	0.196
Total WMH volume (mm^3^), M (Q₁, Q₃)		17621.00 (10597.30, 31688.60)	20764.00 (11675.40, 34971.60)	18104.00 (11879.15, 32140.45)	8678.70 (6023.22, 15873.85)	19636.40 (10528.60, 29837.55)	**0.035**	0.421	0.112	0.257
Periventricular WMH volume (mm^3^), M (Q₁, Q₃)		8022.60 (3590.50, 16490.90)	10032.30 (4401.00, 18780.60)	8022.60 (3882.65, 15445.35)	2687.40 (1388.53, 7738.82)	8272.60 (3778.70, 17962.25)	**0.046**	0.300	0.141	0.403
Juxtacortical WMH volume (mm^3^), M (Q₁, Q₃)		880.20 (329.10, 2160.80)	695.00 (268.60, 1904.20)	1292.80 (428.85, 2489.20)	317.80 (192.05, 601.07)	752.10 (344.65, 1702.70)	**0.001**	0.228	**0.010**	**0.002**
Juxtacortical WMH volume percentage (% of total WM), M (Q₁, Q₃)		0.20 (0.08, 0.45)	0.16 (0.06, 0.40)	0.27 (0.09, 0.54)	0.08 (0.05, 0.13)	0.17 (0.08, 0.39)	**0.002**	0.248	**0.013**	**0.003**
Total CMB count, M (Q₁, Q₃)		1.00 (0.00, 4.00)	3.00 (1.00, 11.00)	1.00 (0.00, 4.00)	0.00 (0.00, 0.75)	1.00 (0.00, 3.00)	**0.008**	**0.003**	**0.044**	0.214
Total CMB volume (mm^3^), M (Q₁, Q₃)		20.01 (0.00, 183.16)	100.85 (5.54, 330.10)	14.95 (0.00, 148.35)	0.00 (0.00, 21.76)	13.95 (0.00, 90.34)	**0.011**	**0.004**	0.065	0.209
BG ePVS score (based on count >10), *n* (%)	0	164 (72.89)	21 (56.8)	93 (75.6)	12 (66.7)	38 (80.9)	0.071	**0.025**	0.413	**0.022**
	1	61 (27.11)	16 (43.2)	30 (24.4)	6 (33.3)	9 (19.1)				
Fazekas score, *n* (%)	1	6 (2.67)	0 (0.0)	1 (0.8)	2 (11.1)	3 (6.4)	NA	0.728	**0.038**	0.685
	2	55 (24.44)	8 (21.6)	30 (24.4)	8 (44.4)	9 (19.1)				
	3	45 (20.00)	7 (18.9)	25 (20.3)	3 (16.7)	10 (21.3)				
	4	98 (43.56)	17 (45.9)	53 (43.1)	4 (22.2)	24 (51.1)				
	5	18 (8.00)	5 (13.5)	11 (8.9)	1 (5.6)	1 (2.1)				
	6	3 (1.33)	0 (0.0)	3 (2.4)	0 (0.0)	0 (0.0)				
Total CSVD Burden										
High TB-CSVD ^a^, *n* (%)	Low	77 (34.22)	6 (16.2)	39 (31.7)	12 (66.7)	20 (42.6)	**0.001**	**0.013**	**0.003**	0.871
	High	148 (65.78)	31 (83.8)	84 (68.3)	6 (33.3)	27 (57.4)				

^a^TB-CSVD was dichotomized as low burden (scores 0–1) and high burden (scores 2–4). Overall P: P for the comparison among the four groups, P^*^: P for the comparison between CSVD with or without MCR (Group A vs Group B + C + D), P^⁑^: P for the comparison between CSVD with or without SCCs (Group A + B vs Group C + D), and P^⁂^: P for the comparison between CSVD with or without slow gait (Group A + C vs Group B + D). Only neuroimaging parameters with at least one comparison showing *P* < 0.05 are presented. Bold values indicate *P* < 0.05. BG, basal ganglia; CSO, centrum semiovale; CSVD, cerebral small vessel disease; ePVS, enlarged perivascular spaces; M, Median; n, number; NA, not available; PVS, perivascular spaces; Q₁, 1st Quartile; Q₃, 3rd Quartile; SCCs, subjective cognitive complaints; TB-CSVD, the total cerebral small vessel disease burden score calculated according to Staals et al. (2014); WMH, white matter hyperintensity.

Neuroimaging comparisons also showed differences across MCR, SCCs, and slow-gait strata. Compared with patients without MCR, patients with MCR had higher lacune count on FLAIR, higher CMB count and volume, and a higher proportion of high TB-CSVD. Patients with SCCs had greater juxtacortical WMH volume and a higher proportion of high TB-CSVD than those without SCCs. In contrast, patients with slow gait, particularly those without SCCs, showed lower juxtacortical WMH volume. Overall, high TB-CSVD was more frequent in patients with MCR and in those with SCCs.

### 3.3 Univariable associations of demographic, clinical and neuroimaging-based parameters with MCR, SCCs and slow gait

Univariable logistic regression analyses identified several demographic, clinical, and neuroimaging parameters that were associated with MCR, SCCs, and slow gait among CSVD patients (Supplementary Tables S1-S4). For MCR among CSVD, significant associated factors included lower SBP (e.g., average SBP: OR, 0.969; 95% CI, 0.943–0.995), poorer functional status (mRS score 1 vs. 0: OR, 2.815; 95% CI, 1.349–5.949), physical inactivity (OR, 7.475; 95% CI, 1.580–39.415), higher basal ganglia perivascular space (BG PVS) counts, and higher TB-CSVD (vs low burden: OR, 3.135; 95% CI, 1.328–8.667). Additionally, dyslipidemia and ICH were associated with higher odds of MCR (OR for dyslipidemia, 2.550; 95% CI, 1.225–5.278; OR for ICH, 16.500; 95% CI, 2.046–338.988).

For SCCs among CSVD, statistically significant factors were younger age (OR, 0.947; 95% CI, 0.907–0.988), fewer years of education (OR, 0.896; 95% CI, 0.810–0.986), higher DBP (e.g., average DBP: OR, 1.041; 95% CI, 1.010–1.076), married status (OR, 3.602; 95% CI, 1.202–11.374), presence of headache (OR, 3.118; 95% CI, 1.256–9.459), greater juxtacortical WMH volume per 1,000 mm^3^ (OR, 1.420; 95% CI, 1.105–1.826), higher Fazekas scores, and higher TB-CSVD (*vs* low burden: OR, 2.478; 95% CI, 1.367–4.516). Diabetes and current alcohol consumption were associated with lower odds of SCCs (OR for diabetes, 0.335; 95% CI, 0.180–0.621; OR for current alcohol consumption, 0.396; 95% CI, 0.184–0.859).

For slow gait among CSVD, significant associated factors included lower systolic and diastolic blood pressure (e.g., average SBP: OR, 0.968; 95% CI, 0.945–0.990), higher income level (high vs. low: OR, 0.190; 95% CI, 0.038–0.720), presence of dyslipidemia (OR, 2.032; 95% CI, 1.062–3.856), worse functional status (mRS score 1 vs. 0: OR, 2.364; 95% CI, 1.241–4.515), lower engagement in moderate- or vigorous-intensity physical activity (e.g., moderate-intensity: OR, 0.402; 95% CI, 0.214–0.749), lower juxtacortical WMH volume per 1,000 mm^3^ (OR, 0.737; 95% CI, 0.569–0.954), and a higher BG-ePVS score (OR, 2.239; 95% CI, 1.166–4.273). Current smoking was associated with lower odds of slow gait (OR, 0.359; 95% CI, 0.119–0.891).

### 3.4 Discrimination of exploratory association-based multivariable models for MCR, SCCs, and slow gait

**Figure 2:**
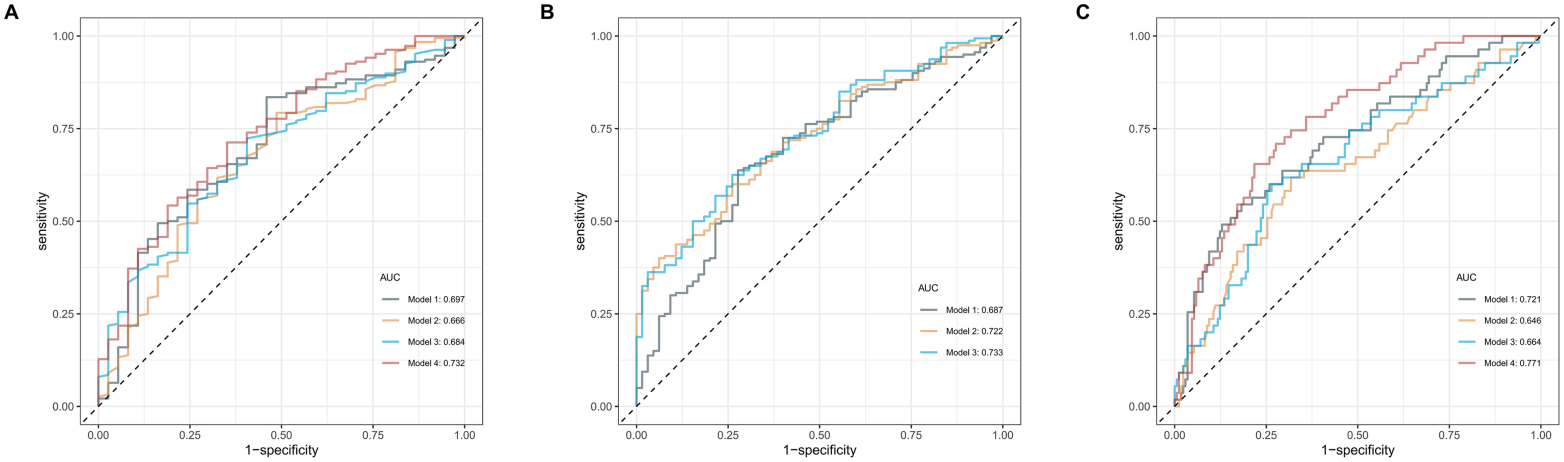
ROC curves of exploratory association-based multivariable models for MCR, SCCs, and slow gait among patients with CSVD. **(A)** Multivariable models for MCR; **(B)** Multivariable models for SCCs; **(C)** Multivariable models for slow gait. **(A)** Model 1: Ref. + Average SBP Model 2: Ref. + Physical inactivity Model 3: Ref. + High TB-CSVD Model 4: Ref. + Average SBP + Physical inactivity + High TB-CSVD. **(B)** Model 1: Ref. + Average DBP + Headache. Model 2: Ref. + Juxtacortical WMH volume (per 1,000 mm^3^) + High TB-CSVD. Model 3: Ref. + Average DBP + Headache + Juxtacortical WMH volume (per 1,000 mm^3^) + High TB-CSVD. **(C)** Model 1: Ref. + Average SBP + Dyslipidemia + mRS. Model 2: Ref. + Moderate-intensity physical activity. Model 3: Ref. + Juxtacortical WMH volume (per 1,000 mm^3^). Model 4: Ref. + Average SBP + Dyslipidemia + mRS + Moderate-intensity physical activity + Juxtacortical WMH volume (per 1,000 mm^3^). The total CSVD burden score (range 0–4) was dichotomized as low (score 0–1) and high (score 2–4); Physical activity intensity was defined as: Light-intensity (1.5–<3 metabolic equivalent of tasks [METs]), Moderate-intensity (3–6 METs), and Vigorous-intensity (≥6 METs); Physical inactivity was defined as no participation in any physical activity. CSVD, cerebral small vessel disease; DBP, diastolic blood pressure; MCR, motoric cognitive risk syndrome; mRS, modified Rankin Scale; Ref., Age, sex, and years of education; SBP, systolic blood pressure; SCCs, subjective cognitive complaints; TB-CSVD, the total CSVD burden calculated following Staals et al. (2014); WMH, white matter hyperintensity.

Exploratory association-based multivariable models were subsequently constructed by integrating baseline demographics (age, sex, and years of education) with selected parameters supported by biological plausibility, clinical relevance, and univariable patterns (Table 3). The corresponding ROC curves for these models are presented in Figure 2 (Panel A for MCR, Panel B for SCCs, and Panel C for slow gait). Because of the limited number of MCR events, the sparse distribution of complete physical inactivity, and the cross-sectional association-based design, these models were interpreted as exploratory rather than as clinically ready screening or prediction tools. We therefore supplemented the primary analyses with Firth penalized logistic regression and bootstrap internal validation, FDR adjustment of univariable screening analyses, and DeLong tests for formal AUC comparisons (Supplementary Tables S5-S8).

**Table 3:** Discrimination of selected exploratory association-based multivariable models for MCR, SCCs, and slow gait among patients with CSVD.

Model	Variable	Apparent AUC (95% CI)	Optimism-corrected AUC^c^	Adjusted OR (95% CI) ^d^	*P*
For MCR among CSVD					
Model 1: Demographic and Clinical Parameters	Ref. + Average SBP	0.697 (0.604–0.791)	0.657	/	/
	Average SBP	/		0.967 (0.939–0.993)	**0.018**
Model 2: Physical Activity	Ref. + Physical inactivity	0.666 (0.569–0.764)	0.628	/	/
	Physical inactivity	/		7.306 (1.488–39.987)	**0.014**
Model 3: CSVD Neuroimaging	Ref. + High TB-CSVD ^a^	0.684 (0.595–0.773)	0.645	/	/
	High TB-CSVD	/		3.185 (1.319–8.929)	**0.016**
Model 4: Demographic and Clinical Parameters, Physical Activity, and CSVD Neuroimaging	Ref. + Average SBP + Physical inactivity + High TB-CSVD	0.732 (0.644–0.820)	0.691	/	/
	Average SBP	/		0.964 (0.936–0.992)	**0.015**
	Physical inactivity	/		7.902 (1.430–50.169)	**0.019**
	High TB-CSVD	/		3.460 (1.385–10.101)	**0.013**
For SCCs among CSVD					
Model 1: Demographic and Clinical Parameters	Ref. + Average DBP + Headache	0.687 (0.611–0.763)	0.658	/	/
	Average DBP	/		1.033 (1.000–1.069)	0.056
	Headache	/		2.808 (1.086–8.775)	**0.048**
Model 2: CSVD Neuroimaging	Ref. + Juxtacortical WMH volume (per 1,000 mm^3^) + High TB-CSVD	0.722 (0.655–0.790)	0.690	/	/
	Juxtacortical WMH volume (per 1,000 mm^3^)	/		1.427 (1.092–1.865)	**0.009**
	High TB-CSVD	/		2.033 (1.071–3.870)	**0.030**
Model 3: Demographic and Clinical Parameters, and CSVD Neuroimaging	Ref. + Average DBP + Headache + Juxtacortical WMH volume (per 1,000 mm^3^) + High TB-CSVD	0.733 (0.666–0.801)	0.696	/	/
	Average DBP	/		1.029 (0.995–1.067)	0.102
	Headache	/		2.601 (0.981–8.264)	0.073
	Juxtacortical WMH volume (per 1,000 mm^3^)	/		1.419 (1.083–1.861)	**0.011**
	High TB-CSVD	/		1.876 (0.969–3.633)	0.061
For slow gait among CSVD					
Model 1: Demographic and Clinical Parameters	Ref. + Average SBP + Dyslipidemia + mRS	0.721 (0.640–0.801)	0.685	/	/
	Average SBP	/		0.962 (0.937–0.986)	**0.003**
	Dyslipidemia	/		2.102 (1.056–4.174)	**0.033**
	mRS	/		2.579 (1.479–4.602)	**<0.001**
Model 2: Physical Activity	Ref. + Moderate-intensity physical activity ^b^	0.646 (0.559–0.733)	0.611	/	/
	Moderate-intensity physical activity	/		0.384 (0.202–0.723)	**0.003**
Model 3: CSVD Neuroimaging	Ref. + Juxtacortical WMH volume (per 1,000 mm^3^)	0.664 (0.579–0.750)	0.624	/	/
	Juxtacortical WMH volume (per 1,000 mm^3^)	/		0.741 (0.572–0.960)	**0.023**
Model 4: Demographic and Clinical Parameters, Physical Activity, and CSVD Neuroimaging	Ref. + Average SBP + Dyslipidemia + mRS + Moderate-intensity physical activity + Juxtacortical WMH volume (per 1,000 mm^3^)	0.770 (0.702–0.839)	0.725	/	/
	Average SBP	/		0.962 (0.937–0.986)	**0.003**
	Dyslipidemia	/		2.147 (1.050–4.391)	**0.035**
	mRS	/		2.464 (1.385–4.466)	**0.002**
	Moderate-intensity physical activity	/		0.518 (0.260–1.033)	0.061
	Juxtacortical WMH volume (per 1,000 mm^3^)	/		0.757 (0.576–0.994)	**0.040**

^a^TB-CSVD was dichotomized as low burden (scores 0–1) and high burden (scores 2–4). ORs for high TB-CSVD indicate comparisons with the low-burden reference group. ^b^Physical activity intensity was defined as: Light-intensity (1.5–<3 metabolic equivalent of tasks [METs]), Moderate-intensity (3–6 METs), and Vigorous-intensity (≥6 METs). ^c^Optimism-corrected AUCs were estimated using bootstrap internal validation with 1,000 resamples and were presented as point estimates. ^d^Adjusted ORs were obtained from multivariable logistic regression models adjusted for age, sex, years of education, and the listed model-specific variables. Univariable ORs for the corresponding candidate variables are provided in Supplementary Tables S1–S4. Absolute lesion volumes were rescaled per 1,000 mm^3^ in logistic regression models to improve interpretability of ORs. Bold values indicate *P* < 0.05. CSVD, cerebral small vessel disease; DBP, diastolic blood pressure; MCR, motoric cognitive risk syndrome; mRS, modified Rankin Scale; Ref.: Age, sex, and years of education; SBP, systolic blood pressure; SCCs, subjective cognitive complaints; TB-CSVD, the total cerebral small vessel disease burden score calculated according to Staals et al. (2014); WMH, white matter hyperintensity.

For MCR in CSVD, Model 1 (demographics + average SBP) yielded an apparent AUC of 0.697 (95% CI, 0.604–0.791), with an optimism-corrected AUC of 0.657. Model 2 (demographics + physical inactivity) and Model 3 (demographics + high TB-CSVD) showed apparent AUCs of 0.666 and 0.684, respectively, with optimism-corrected AUCs of 0.628 and 0.645. The comprehensive Model 4, combining demographics, average SBP, physical inactivity, and high TB-CSVD, demonstrated an apparent AUC of 0.732 (95% CI, 0.644–0.820), which decreased to 0.691 after bootstrap optimism correction. The EPV for this model was 6.17. In Firth penalized logistic regression, the associations remained directionally consistent for average SBP (OR, 0.967; 95% CI, 0.939–0.993), physical inactivity (OR, 6.888; 95% CI, 1.408–38.084), and high TB-CSVD (OR, 3.143; 95% CI, 1.306–8.680), although the estimate for physical inactivity remained imprecise.

For SCCs in CSVD, Model 1 (demographics + average DBP + headache) achieved an apparent AUC of 0.687 (95% CI, 0.611–0.763), with an optimism-corrected AUC of 0.658. Model 2, incorporating neuroimaging markers (demographics + juxtacortical WMH volume per 1,000 mm^3^ + high TB-CSVD), showed an apparent AUC of 0.722 (95% CI, 0.655–0.790), with an optimism-corrected AUC of 0.690. The combined Model 3 (demographics + average DBP + headache + juxtacortical WMH volume per 1,000 mm^3^ + high TB-CSVD) showed an apparent AUC of 0.733 and an optimism-corrected AUC of 0.696.

For slow gait in CSVD, Model 1 (demographics + average SBP + dyslipidemia + mRS) yielded an apparent AUC of 0.721 (95% CI, 0.640–0.801), with an optimism-corrected AUC of 0.685. Model 2 (demographics + moderate-intensity physical activity) and Model 3 (demographics + juxtacortical WMH volume per 1,000 mm^3^) showed apparent AUCs of 0.646 and 0.664, respectively, with optimism-corrected AUCs of 0.611 and 0.624. The integrative Model 4, which combined demographics; clinical parameters including average SBP, dyslipidemia and mRS; moderate-intensity physical activity; and juxtacortical WMH volume per 1,000 mm^3^ as the CSVD neuroimaging feature, showed an apparent AUC of 0.770 and an optimism-corrected AUC of 0.725.

Formal pairwise DeLong tests are presented in Supplementary Table S8. For MCR, the comprehensive Model 4 showed a higher apparent AUC than the demographic reference model (0.732 vs. 0.636; difference, 0.097; DeLong *p* = 0.036), but did not significantly outperform Model 1 (*p* = 0.321), Model 2 (*p* = 0.098), or Model 3 (*p* = 0.115). For SCCs, the combined Model 3 showed a higher apparent AUC than the demographic reference model (0.733 vs. 0.654; difference, 0.079; DeLong *p* = 0.032), but not than the clinical model (*p* = 0.092) or neuroimaging model (*p* = 0.564). For slow gait, the combined Model 4 showed higher apparent AUCs than the demographic reference model and all single-domain models (all DeLong *p* ≤ 0.033). For multiplicity-aware interpretation of the univariable screening analyses, Benjamini-Hochberg FDR correction was applied separately within each outcome across 87 predictor-level tests. Among variables with raw *p* < 0.05, no MCR or slow-gait variable remained significant after FDR correction, whereas diabetes for SCCs remained significant (FDR-adjusted *p* = 0.047). Therefore, the univariable screening findings were interpreted as exploratory and hypothesis-generating.

## 4 Discussion

This exploratory cross-sectional study preliminarily investigated factors associated with MCR and its components in 225 Chinese patients with CSVD who did not meet the MoCA-based screening criterion for possible dementia. The prevalence of MCR in this population was 16.4%. Multivariable models integrating demographic, clinical, and neuroimaging factors achieved AUCs ranging from 0.73 to 0.77; after bootstrap internal validation, optimism-corrected AUCs ranged from 0.69 to 0.73, indicating modest exploratory discrimination rather than clinically ready prediction.

The observed MCR prevalence of 16.4% in this CSVD cohort study is higher than the upper limit reported for general older populations (5.3–15.5%)(Marquez et al., 2022). This may be related to the underlying CSVD pathology. CSVD may simultaneously affect cognitive and motor pathways (Huang et al., 2024) through mechanisms such as hypoperfusion, blood–brain barrier disruption, and neuroinflammation (Gurol et al., 2020; Tuladhar et al., 2020; Ter Telgte and Duering, 2024). The prevalence of MCR differs from the 37.3% reported in an Indian CSVD study (Wang et al., 2016). This discrepancy might be partly explained by differences in demographic and clinical characteristics (Iqbal et al., 2022), including differences in vascular risk profiles, functional status, gait assessment procedures, and study-specific inclusion criteria. Other factors, including genetic background and healthcare accessibility, may also contribute to such discrepancy (Mullin et al., 2023; Martins et al., 2025).

Our analysis revealed complex relationships regarding SCCs. Juxtacortical WMH volume remained associated with SCCs across models. This finding is broadly consistent with emerging evidence that global and regional WMH burden may contribute to cognitive-motor vulnerability and MCR-related outcomes in older adults, although the present SCC measure was limited to a single-item memory complaint (Vazquez et al., 2026). The high TB-CSVD showed a positive association with both SCCs and MCR, reinforcing the concept that an accumulating load of CSVD lesions contributes to cognitive-motor vulnerability. The slight attenuation of these neuroimaging effects in the final combined model may reflect shared variance between imaging markers, such as juxtacortical WMH volume and the high TB-CSVD.

For slow gait, the associations with lower average SBP and lower juxtacortical WMH volume should be interpreted cautiously as exploratory observations rather than as direct mechanistic effects. In descriptive comparisons, patients with slow gait had a lower median average SBP than those without slow gait, but poorer functional status, dyslipidemia, reduced participation in moderate- or vigorous-intensity physical activity, and higher BG-ePVS were also associated with slow gait. Therefore, the lower-SBP finding may reflect clinical heterogeneity, frailty or functional status, treatment-selection effects, or cerebral hemodynamic vulnerability rather than a protective effect of higher blood pressure. The relationship between blood pressure, cerebral perfusion, and mobility in older adults is complex and may vary according to age and physiological reserve (Windham et al., 2023). Similarly, the inverse association between juxtacortical WMH volume and slow gait should not be interpreted as evidence that lower WMH burden is biologically linked to slower gait. In our data, total WMH volume did not differ significantly between patients with and without slow gait, and the lowest juxtacortical WMH volume was observed in the small subgroup with slow gait but without SCCs (Group C; *n =* 18). Existing evidence generally supports an association between greater CSVD or global WMH burden and gait impairment, but regional WMH associations are less consistent and may vary across cohorts, imaging methods, and clinical contexts (Doi et al., 2022; Sharma et al., 2023). Therefore, this regional inverse association may reflect subgroup heterogeneity, residual confounding, or chance findings related to multiple regional comparisons. Because sufficiently complete antihypertensive medication data, cerebral perfusion data, brain atrophy measures, and longitudinal assessments were unavailable, we could not formally test medication-related confounding, hemodynamic mechanisms, mediation, or non-linear effects. These findings should therefore be regarded as exploratory and hypothesis-generating.

Multi-domain models showed numerically higher apparent AUCs compared to single-domain models in our population. However, DeLong tests indicated that the comprehensive MCR and SCCs models were significantly higher than the demographic reference models but did not significantly outperform the corresponding single-domain models; only the slow-gait combined model showed statistically higher AUCs than both the demographic reference model and the single-domain models. The comprehensive models for MCR combined a vascular factor (lower SBP), a lifestyle factor (physical inactivity), and a neuroimaging marker (TB-CSVD). High TB-CSVD was positively associated with MCR, which is consistent with the descriptive findings and with the biological plausibility that accumulated CSVD burden may disrupt cognitive-motor networks (Wardlaw et al., 2019). Physical inactivity may exacerbate vascular and muscle dysfunction (El Assar et al., 2022). However, complete physical inactivity was rare, and the corresponding confidence intervals remained wide even after Firth penalized regression. The association between lower average SBP and MCR should be interpreted cautiously in this cross-sectional analysis and may reflect reverse causation, treatment effects, or unmeasured medication use. Therefore, these models should be considered exploratory association-based multivariable models rather than definitive prediction models or clinically ready screening tools.

These findings have several potential clinical implications, but should be interpreted primarily as hypothesis-generating. First, the exploratory models suggest that routinely available demographic, clinical, lifestyle-related, and neuroimaging variables may contribute to future risk-stratification research, but the present models are not suitable for direct clinical decision-making. Second, the results support the value of comprehensive cognitive-motor assessment in CSVD, including objective gait speed measurement and careful characterization of cognitive complaints. Third, the observed signals, including physical inactivity and high TB-CSVD, require confirmation in larger, prospective, multicenter cohorts before they can be considered robust correlates or used for clinical stratification.

This study has several limitations. First, its cross-sectional design precludes causal inference, temporality, and prediction of future MCR or dementia. In particular, the association between physical inactivity and MCR may be bidirectional, because physical inactivity may contribute to motor-cognitive vulnerability, whereas MCR or slow gait may also reduce physical activity. Longitudinal studies are needed to clarify temporality. Second, physical activity was self-reported and may be affected by recall bias and social desirability bias. Third, although the parent cohort was a national CSVD registry, the present analysis was restricted to the cognitive subgroup with complete SCC and gait assessments, mainly from Beijing Tiantan Hospital. Therefore, the findings should be interpreted as registry-derived but not fully representative of all centers in the national registry, and external validation in broader multicenter CSVD cohorts is needed. Fourth, although all participants were clinically assessed by neurologists and patients with diagnosed Parkinson’s disease, parkinsonism, or other neurodegenerative diseases were excluded, MCR is a cross-disease clinical risk phenotype rather than a disease-specific diagnosis. Structured screening for prodromal or early-stage Parkinson’s disease was not systematically performed; therefore, unrecognized early-stage Parkinson’s disease cannot be completely excluded and may have contributed to slow gait or cognitive complaints in a small proportion of patients. Fifth, SCCs were assessed using a single memory-related item from the GDS-15. Although this item has been used in previous MCR studies and was feasible within a registry-based assessment, SCCs in this study should be interpreted as single-item memory complaints rather than as a psychometrically comprehensive assessment of multidimensional SCD. This approach may have limited construct validity and may be susceptible to affective contamination. Because cleaned and harmonized data on total depressive symptoms, anxiety, or apathy were unavailable, we could not adjust for these neuropsychiatric symptoms, and misclassification of SCCs and, consequently, the MCR phenotype remains possible. Sixth, dementia exclusion relied on an education-adjusted MoCA-full screening threshold rather than comprehensive clinical dementia adjudication. This pragmatic approach helped exclude participants with possible dementia but may affect phenotype classification and generalizability, particularly because MoCA thresholds vary across language versions, educational backgrounds, and clinical settings. Seventh, although candidate variables were selected using both clinical relevance and univariable statistical evidence, the modeling process remained partly data-informed and exploratory. A large number of univariable comparisons were performed, and most conventional univariable associations did not remain significant after FDR adjustment; therefore, these findings should not be interpreted as confirmatory or as robust correlates. Eighth, the number of MCR events was limited and complete physical inactivity was sparse, leading to potential overfitting and imprecise estimates. Although Firth penalized logistic regression and bootstrap internal validation were added, these approaches reduce but do not eliminate small-sample and sparse-data concerns. Therefore, the models should not be interpreted as providing stable multivariable inference or clinically ready prediction. The models were developed and internally evaluated in the same cohort and require validation in larger independent cohorts before clinical implementation. Finally, standardized quantitative or harmonized visual measures of brain atrophy were unavailable in the final analytical dataset, and paired pre-consensus imaging ratings were not retained. Therefore, we could not evaluate the contribution of global or regional atrophy to MCR and its components, nor could we retrospectively calculate inter-rater reliability statistics such as *κ* values for the visual MRI markers. Several unexpected associations, including lower SBP and lower juxtacortical WMH volume in relation to slow gait, could not be fully explored because sufficiently complete antihypertensive medication information, non-linear modeling, mediation analyses, and longitudinal assessments were unavailable. These findings should therefore be regarded as exploratory and hypothesis-generating, rather than as a confirmatory study establishing robust correlates, temporality, or prediction.

In conclusion, this exploratory cross-sectional study observed associations between selected demographic, clinical, lifestyle-related, and neuroimaging variables with MCR and its components in CSVD patients without possible dementia based on MoCA screening. High TB-CSVD showed an association with higher odds of MCR, while physical inactivity also showed a positive but imprecise association because of sparse exposure. Lower average SBP was observed in association with MCR and should be interpreted cautiously. Given the small number of MCR events, pragmatic single-item SCC assessment, cross-sectional design, and limited persistence of findings after FDR correction, these results should be considered hypothesis-generating. Prospective external validation is required before any clinical application.

## Data Availability

The data underlying this article will be shared by the corresponding author upon reasonable request, subject to institutional and ethical approval.

## References

[ref1] Ainsworth B. E. Haskell W. L. Herrmann S. D. Meckes N. Bassett D. R. Tudor-Locke C. . (2011). 2011 compendium of physical activities: a second update of codes and MET values. Med. Sci. Sports Exerc. 43, 1575–1581. doi: 10.1249/MSS.0b013e31821ece12, 21681120

[ref2] Azevedo L. Marques A. Silva-Júnior G. A. de Santos G. S. S. Faria L. P. Taveira M. L. . (2025). Motoric cognitive risk syndrome: prevalence and associated sociodemographic and clinical factors in primary care settings in Brazil. Alzheimers Dement. 21:e70903. doi: 10.1002/alz.70903, 41246867 PMC12620998

[ref3] Bai A. Bai W. Ju H. Xu W. Lin Z. (2022). Motoric cognitive risk syndrome as a predictor of incident disability: a 7 year follow-up study. Front. Aging Neurosci. 14:972843. doi: 10.3389/fnagi.2022.972843, 36158535 PMC9493455

[ref4] Doi T. Nakakubo S. Tsutsumimoto K. Kurita S. Kiuchi Y. Nishimoto K. . (2022). The association of white matter hyperintensities with motoric cognitive risk syndrome. Cereb Circ Cogn Behav 3:100150. doi: 10.1016/j.cccb.2022.100150 36324398 PMC9616382

[ref5] Duering M. Biessels G. J. Brodtmann A. Chen C. Cordonnier C. de Leeuw F. E. . (2023). Neuroimaging standards for research into small vessel disease-advances since 2013. Lancet Neurol. 22, 602–618. doi: 10.1016/s1474-4422(23)00131-x, 37236211

[ref6] El Assar M. Álvarez-Bustos A. Sosa P. Angulo J. Rodríguez-Mañas L. (2022). Effect of physical activity/exercise on oxidative stress and inflammation in muscle and vascular aging. Int. J. Mol. Sci. 23:8713. doi: 10.3390/ijms23158713, 35955849 PMC9369066

[ref7] Fazekas F. Chawluk J. B. Alavi A. Hurtig H. I. Zimmerman R. A. (1987). MR signal abnormalities at 1.5 T in Alzheimer's dementia and normal aging. AJR Am. J. Roentgenol. 149, 351–356. doi: 10.2214/ajr.149.2.351 3496763

[ref8] Gurol M. E. Sacco R. L. McCullough L. D. (2020). Multiple faces of cerebral small vessel diseases. Stroke 51, 9–11. doi: 10.1161/strokeaha.119.027969, 31752615 PMC7590926

[ref9] Hagiwara A. Kamio S. Kikuta J. Nakaya M. Uchida W. Fujita S. . (2025). Decoding brain development and aging: pioneering insights from MRI techniques. Investig. Radiol. 60, 162–174. doi: 10.1097/rli.0000000000001120, 39724579 PMC11801466

[ref10] Huang C. Wu B. Zhang C. Wei Z. Su L. Zhang J. . (2024). Motoric cognitive risk syndrome as a predictor of adverse health outcomes: a systematic review and Meta-analysis. Gerontology 70, 669–688. doi: 10.1159/000538314, 38697041

[ref11] Iqbal K. Hasanain M. Ahmed J. Iqbal A. Rathore S. S. Monis A. . (2022). Association of Motoric Cognitive Risk Syndrome with cardiovascular and noncardiovascular factors: a systematic review and Meta-analysis. J. Am. Med. Dir. Assoc. 23, 810–822. doi: 10.1016/j.jamda.2021.11.035, 34973959

[ref12] Jessen F. Amariglio R. E. Buckley R. F. van der Flier W. M. Han Y. Molinuevo J. L. . (2020). The characterisation of subjective cognitive decline. Lancet Neurol. 19, 271–278. doi: 10.1016/s1474-4422(19)30368-0, 31958406 PMC7062546

[ref13] Jessen F. Amariglio R. E. van Boxtel M. Breteler M. Ceccaldi M. Chételat G. . (2014). A conceptual framework for research on subjective cognitive decline in preclinical Alzheimer's disease. Alzheimers Dement. 10, 844–852. doi: 10.1016/j.jalz.2014.01.001, 24798886 PMC4317324

[ref14] Kim S. E. Kim H. J. Jang H. Weiner M. W. DeCarli C. Na D. L. . (2022). Interaction between Alzheimer's disease and cerebral small vessel disease: a review focused on neuroimaging markers. Int. J. Mol. Sci. 23:490. doi: 10.3390/ijms231810490, 36142419 PMC9499680

[ref15] Lau H. Mat Ludin A. F. Shahar S. Badrasawi M. Clark B. C. (2019). Factors associated with motoric cognitive risk syndrome among low-income older adults in Malaysia. BMC Public Health 19:462. doi: 10.1186/s12889-019-6869-z, 31196017 PMC6565538

[ref16] Lim N. E. Yeo B. S. Y. Lee R. S. Lim J. X. Chan Y. H. Kandiah N. . (2024). Motoric cognitive risk syndrome as a predictive factor of cognitive impairment and dementia - a systematic review and meta-analysis. Ageing Res. Rev. 101:102470. doi: 10.1016/j.arr.2024.102470 39245075

[ref17] Marquez I. Garcia-Cifuentes E. Velandia F. R. Iragorri A. Saavedra A. M. Borda M. G. . (2022). Motoric cognitive risk syndrome: prevalence and cognitive performance. A cross-sectional study. Lancet Reg. Health Am. 8:100162. doi: 10.1016/j.lana.2021.100162, 36778728 PMC9904094

[ref18] Martins J. P. Fukushima F. B. Benatti L. N. Bazan R. Silva K. Vidal E. I. O. (2025). Prevalence of motoric cognitive risk syndrome among older adults in Brazil and evaluation of effect modification by race. J. Alzheimer's Dis 103, 785–796. doi: 10.1177/13872877241300296, 39584365

[ref19] Mullin D. S. Stirland L. E. Russ T. C. Luciano M. Muniz-Terrera G. (2023). Socioeconomic status as a risk factor for motoric cognitive risk syndrome in a community-dwelling population: a longitudinal observational study. Eur. J. Neurol. 30, 1191–1199. doi: 10.1111/ene.15731 36755198

[ref20] Nasreddine Z. S. Phillips N. A. Bédirian V. Charbonneau S. Whitehead V. Collin I. . (2005). The Montreal cognitive assessment, MoCA: a brief screening tool for mild cognitive impairment. J. Am. Geriatr. Soc. 53, 695–699. doi: 10.1111/j.1532-5415.2005.53221.x, 15817019

[ref21] Quinn T. J. Dawson J. Walters M. R. Lees K. R. (2009). Reliability of the modified Rankin scale: a systematic review. Stroke 40, 3393–3395. doi: 10.1161/strokeaha.109.557256, 19679846

[ref22] Sachdev P. S. Bentvelzen A. C. Kochan N. A. Jiang J. Hosoki S. Koncz R. . (2025). Revised diagnostic criteria for vascular cognitive impairment and dementia-the VasCog-2-WSO criteria. JAMA Neurol. 82, 1103–1112. doi: 10.1001/jamaneurol.2025.3242, 40955506 PMC12441927

[ref23] Saks D. G. Sachdev P. S. (2025). Monogenic causes of cerebral small vessel disease- models for vascular cognitive impairment and dementia? Curr. Opin. Psychiatry 38, 112–118. doi: 10.1097/yco.0000000000000978, 39840612 PMC11789596

[ref24] Sathyan S. Ayers E. Blumen H. Weiss E. F. Adhikari D. Stimmel M. . (2023). Epidemiology of motoric cognitive risk syndrome in the Kerala einstein study: protocol for a prospective cohort study. JMIR Res Protoc 12:e49933. doi: 10.2196/49933, 37590054 PMC10472178

[ref25] Sharma B. Wang M. McCreary C. R. Camicioli R. Smith E. E. (2023). Gait and falls in cerebral small vessel disease: a systematic review and meta-analysis. Age Ageing 52:011. doi: 10.1093/ageing/afad011, 37000039 PMC10064981

[ref26] Shen S. Zeng X. Xu L. Chen L. Liu Z. Chu J. . (2020). Association between motoric cognitive risk syndrome and frailty among older Chinese adults. BMC Geriatr. 20:110. doi: 10.1186/s12877-020-01511-0, 32192446 PMC7081673

[ref27] Staals J. Makin S. D. Doubal F. N. Dennis M. S. Wardlaw J. M. (2014). Stroke subtype, vascular risk factors, and total MRI brain small-vessel disease burden. Neurology 83, 1228–1234. doi: 10.1212/wnl.0000000000000837, 25165388 PMC4180484

[ref28] Studenski S. Perera S. Patel K. Rosano C. Faulkner K. Inzitari M. . (2011). Gait speed and survival in older adults. JAMA 305, 50–58. doi: 10.1001/jama.2010.1923, 21205966 PMC3080184

[ref29] Ter Telgte A. Duering M. (2024). Cerebral small vessel disease: advancing knowledge with neuroimaging. Stroke 55, 1686–1688. doi: 10.1161/strokeaha.123.044294, 38328947

[ref30] Tian Y. Jiang C. Wang M. Cai R. Zhang Y. He Z. . (2016). BMI, leisure-time physical activity, and physical fitness in adults in China: results from a series of national surveys, 2000-14. Lancet Diabetes Endocrinol. 4, 487–497. doi: 10.1016/s2213-8587(16)00081-4, 27133172

[ref31] Tuladhar A. M. Tay J. van Leijsen E. Lawrence A. J. van Uden I. W. M. Bergkamp M. . (2020). Structural network changes in cerebral small vessel disease. J. Neurol. Neurosurg. Psychiatry 91, 196–203. doi: 10.1136/jnnp-2019-321767, 31744851

[ref32] Twait E. L. Min B. Beran M. Vonk J. M. J. Geerlings M. I. (2023). The cross-sectional association between amyloid burden and white matter hyperintensities in older adults without cognitive impairment: a systematic review and meta-analysis. Ageing Res. Rev. 88:101952. doi: 10.1016/j.arr.2023.101952, 37178806

[ref33] Vazquez J. P. Allali G. Beauchet O. Callisaya M. Doi T. Kumar V. P. . (2026). Cerebral small vessel disease unveils a vascular pathway to motoric cognitive risk in aging. J. Alzheimer's Dis 109, 1211–1219. doi: 10.1177/13872877251405448, 41428469

[ref34] Verghese J. Annweiler C. Ayers E. Barzilai N. Beauchet O. Bennett D. A. . (2014). Motoric cognitive risk syndrome: multicountry prevalence and dementia risk. Neurology 83, 718–726. doi: 10.1212/wnl.0000000000000717, 25031288 PMC4150127

[ref35] Verghese J. Wang C. Bennett D. A. Lipton R. B. Katz M. J. Ayers E. (2019). Motoric cognitive risk syndrome and predictors of transition to dementia: a multicenter study. Alzheimers Dement. 15, 870–877. doi: 10.1016/j.jalz.2019.03.011, 31164315 PMC6646063

[ref36] Verghese J. Wang C. Lipton R. B. Holtzer R. (2013). Motoric cognitive risk syndrome and the risk of dementia. J. Gerontol. A Biol. Sci. Med. Sci. 68, 412–418. doi: 10.1093/gerona/gls191, 22987797 PMC3593614

[ref37] Wang N. Allali G. Kesavadas C. Noone M. L. Pradeep V. G. Blumen H. M. . (2016). Cerebral small vessel disease and motoric cognitive risk syndrome: results from the Kerala-einstein study. J. Alzheimer's Dis 50, 699–707. doi: 10.3233/jad-150523, 26757037 PMC5292924

[ref38] Wardlaw J. M. Smith C. Dichgans M. (2019). Small vessel disease: mechanisms and clinical implications. Lancet Neurol. 18, 684–696. doi: 10.1016/s1474-4422(19)30079-1, 31097385

[ref39] WHO (2020). WHO Guidelines on Physical Activity and Sedentary Behaviour. Geneva: World Health Organization.

[ref40] Windham B. G. Griswold M. E. Ranadive R. Sullivan K. J. Mosley T. H. Mielke M. M. . (2023). Relationships of cerebral perfusion with gait speed across systolic blood pressure levels and age: a cohort study. J. Gerontol. A Biol. Sci. Med. Sci. 78, 514–520. doi: 10.1093/gerona/glac120, 35640170 PMC9977228

[ref41] Xiang K. Liu Y. Sun L. (2021). Motoric cognitive risk syndrome: symptoms, pathology, diagnosis, and recovery. Front. Aging Neurosci. 13:728799. doi: 10.3389/fnagi.2021.728799, 35185512 PMC8847709

[ref42] Yesavage J. A. Brink T. L. Rose T. L. Lum O. Huang V. Adey M. . (1982). Development and validation of a geriatric depression screening scale: a preliminary report. J. Psychiatr. Res. 17, 37–49. doi: 10.1016/0022-3956(82)90033-4, 7183759

[ref43] Zeng W. Zhang L. Feng B. Li H. Wang D. Zheng Z. . (2021). Association between sleep disturbance with motoric cognitive risk syndrome in Chinese older adults. Eur. J. Neurol. 28, 1470–1478. doi: 10.1111/ene.14681, 33316114

[ref44] Zhang L. Feng B. L. Wang C. Y. Zhang Y. Lin P. Zhang Y. L. . (2020). Prevalence and factors associated with motoric cognitive risk syndrome in community-dwelling older Chinese: a cross-sectional study. Eur. J. Neurol. 27, 1137–1145. doi: 10.1111/ene.14266, 32301557

[ref45] Zhao M. Hu G. Lu Y. Yang Q. Chen X. Wang D. . (2024). Association between electroencephalogram alpha-band oscillations and executive and processing functions in patients with cerebral small vessel diseases. J. Cereb. Blood Flow Metab. 44, 1302–1315. doi: 10.1177/0271678x241254677, 38749908 PMC11542127

