# Supplementary material for: Clinical and neuroimaging correlates of motoric cognitive risk syndrome in cerebral small vessel disease: a cross-sectional study
Source: Front Aging Neurosci. 2026 Jun 11;18:1853965. doi: 10.3389/fnagi.2026.1853965 (PMC13294206; doi:10.3389/fnagi.2026.1853965)
Supplement: Supplementary file 1 [file Data_Sheet_1.pdf]

Supplementary Material

Table S1. Univariable associations of selected demographic, clinical, and neuroimaging parameters with MCR, SCCs, and slow gait among patients with CSVD. 2

Table S2. Univariable associations of demographic, clinical, and neuroimaging parameters with MCR among patients with CSVD. 6

Table S3. Univariable associations of demographic, clinical, and neuroimaging parameters with SCCs among patients with CSVD. 11

Table S4. Univariable associations of demographic, clinical, and neuroimaging parameters with slow gait among patients with CSVD. 17

Table S5. Firth penalized logistic regression estimates for exploratory multivariable models and physical-activity sensitivity analyses.......... 22

Table S6. Bootstrap internal validation and optimism-corrected AUCs for exploratory multivariable models. 25

Table S7. Sparse-exposure distributions for key binary variables used in multivariable and sensitivity analyses..... 27

Table S8. Pairwise DeLong comparisons of apparent AUCs between exploratory multivariable models. 28

Table S1. Univariable associations of selected demographic, clinical, and neuroimaging parameters with MCR, SCCs, and slow gait among patients with CSVD.

Parameter	Level	OR (95% CI) [a]	*P*	AUC (95% CI)
**For MCR among CSVD**				
Demographic and Clinical Parameters				
Left SBP		0.965 (0.940-0.989)	**0.007**	0.648 (0.557-0.739)
Average SBP		0.969 (0.943-0.995)	**0.023**	0.626 (0.537-0.716)
Dyslipidemia		2.550 (1.225-5.278)	**0.011**	0.605 (0.518-0.692)
ICH		16.500 (2.046-338.988)	**0.017**	0.538 (0.493-0.583)
mRS	Overall (Reference: 0)	/	**0.019**	0.624 (0.535-0.712)
	1	2.815 (1.349-5.949)	**0.006**	/
	2	2.667 (0.370-12.759)	0.253	/
Physical Activity				
Physical inactivity [b]		7.475 (1.580-39.415)	**0.011**	0.546 (0.495-0.598)
CSVD Neuroimaging Markers				
Left BG PVS count		1.030 (1.002-1.059)	**0.033**	0.621 (0.529-0.713)
Right BG PVS count		1.030 (1.001-1.061)	**0.044**	0.611 (0.511-0.712)
Overall BG PVS count		1.017 (1.002-1.032)	**0.029**	0.622 (0.526-0.717)
BG ePVS score		2.421 (1.153-5.026)	**0.018**	0.597 (0.510-0.683)
High TB-CSVD [c]	(Reference: Low)	3.135 (1.328-8.667)	**0.015**	0.608 (0.538-0.677)
**For SCCs among CSVD**				
Demographic and Clinical Parameters				

Parameter	Level	OR (95% CI) [a]	*P*	AUC (95% CI)
Age		0.947 (0.907-0.988)	**0.012**	0.393 (0.314-0.473)
Years of education		0.896 (0.810-0.986)	**0.027**	0.413 (0.335-0.491)
Right DBP		1.036 (1.006-1.068)	**0.021**	0.606 (0.524-0.688)
Left DBP		1.038 (1.008-1.070)	**0.015**	0.618 (0.535-0.702)
Average DBP		1.041 (1.010-1.076)	**0.012**	0.617 (0.534-0.699)
Married		3.602 (1.202-11.374)	**0.023**	0.543 (0.500-0.586)
Current alcohol consumption		0.396 (0.184-0.859)	**0.018**	0.438 (0.381-0.495)
Diabetes		0.335 (0.180-0.621)	**<0.001**	0.383 (0.314-0.452)
Headache		3.118 (1.256-9.459)	**0.024**	0.565 (0.519-0.610)
CSVD Neuroimaging Markers				
Juxtacortical WMH volume (per 1,000 mm³)		1.420 (1.105-1.826)	**0.006**	0.610 (0.532-0.688)
Juxtacortical WMH volume percentage (% of total WM)		4.968 (1.704-17.582)	**0.007**	0.606 (0.528-0.684)
Fazekas score	Overall (Reference: 1)	/	**0.022**	0.584 (0.507-0.662)
	2	11.176 (1.641-222.989)	**0.033**	/
	3	12.308 (1.765-248.290)	**0.028**	/
	4	12.500 (1.906-245.260)	**0.024**	/
	5	40.000 (4.059-1042.111)	**0.005**	/
	6	28789064.297 (0.000-NA)	0.984	/
Fazekas grade	Overall (Reference: 1)	/	**0.045**	0.575 (0.507-0.643)
	2	1.403 (0.737-2.642)	0.296	/
	3	5.359 (1.377-35.632)	**0.034**	/

Parameter	Level	OR (95% CI) [a]	*P*	AUC (95% CI)
High TB-CSVD	(Reference: Low)	2.478 (1.367-4.516)	**0.003**	0.606 (0.535-0.676)
**For slow gait among CSVD**				
Demographic and Clinical Parameters				
Right SBP		0.976 (0.955-0.997)	**0.026**	0.604 (0.524-0.684)
Left SBP		0.967 (0.945-0.987)	**0.003**	0.643 (0.564-0.721)
Left DBP		0.969 (0.939-0.999)	**0.047**	0.599 (0.515-0.683)
Average SBP		0.968 (0.945-0.990)	**0.006**	0.632 (0.554-0.710)
Average income level [d]	Overall (Reference: Low)	/	**0.044**	0.418 (0.353-0.482)
	Moderate	0.455 (0.197-1.081)	0.068	/
	High	0.190 (0.038-0.720)	**0.022**	/
Current smoking		0.359 (0.119-0.891)	**0.043**	0.437 (0.387-0.486)
Dyslipidemia		2.032 (1.062-3.856)	**0.031**	0.576 (0.503-0.649)
mRS	Overall (Reference: 0)	/	**0.009**	0.616 (0.539-0.692)
	1	2.364 (1.241-4.515)	**0.009**	/
	2	4.538 (1.015-20.353)	**0.041**	/
Physical Activity				
Vigorous-intensity physical activity [e]		0.356 (0.147-0.768)	**0.013**	0.411 (0.352-0.470)
Moderate-intensity physical activity		0.402 (0.214-0.749)	**0.004**	0.607 (0.532-0.682)
CSVD Neuroimaging Markers				
Juxtacortical WMH volume (per 1,000 mm^3)		0.737 (0.569-0.954)	**0.021**	0.638 (0.554-0.723)
Juxtacortical WMH volume percentage (% of total WM)		0.287 (0.081-0.828)	**0.035**	0.631 (0.545-0.717)
BG ePVS score		2.239 (1.166-4.273)	**0.015**	0.585 (0.513-0.658)

Note: a: Absolute lesion volumes were rescaled per 1,000 mm³ in logistic regression models to improve interpretability of ORs. b: Physical inactivity was defined as no participation in any physical activity. c: TB-CSVD was dichotomized as low burden (scores 0–1) and high burden (scores 2–4). ORs for high TB-CSVD indicate comparisons with the low-burden reference group. d: Average income level was defined as: Low (monthly household income per capita <2000 CNY), Moderate (monthly household income per capita 2000-<6000 CNY), and High (monthly household income per capita ≥6000 CNY). e: Physical activity intensity was defined as: Light-intensity (1.5–<3 metabolic equivalent of tasks [METs]), Moderate-intensity (3–6 METs), and Vigorous-intensity (≥6 METs).
Abbreviations: BG, basal ganglia; CSVD, cerebral small vessel disease; DBP, diastolic blood pressure; ICH, intracerebral hemorrhage; MCR, motoric cognitive risk syndrome; mRS, modified Rankin Scale; PVS, perivascular spaces; SBP, systolic blood pressure; SCCs, subjective cognitive complaints; TB-CSVD, the total cerebral small vessel disease burden score calculated according to Staals et al.; WM, white matter; WMH, white matter hyperintensity.

Table S2. Univariable associations of demographic, clinical, and neuroimaging parameters with MCR among patients with CSVD.

Variable	Level	OR (95% CI) [a]	*P*	AUC (95% CI)
**Demographic and Clinical Parameters**				
Demographics				
Age		0.956 (0.903-1.008)	0.105	0.583 (0.485-0.681)
Female		1.314 (0.635-2.678)	0.454	0.533 (0.445-0.621)
Years of education		0.896 (0.795-1.006)	0.065	0.573 (0.478-0.668)
Married		9.05×10^{7} (0.000-NA)	0.988	0.537 (0.518-0.556)
Solitary		2.455 (0.461-45.415)	0.395	0.518 (0.487-0.550)
Insured		0.718 (0.038-4.212)	0.760	0.505 (0.475-0.535)
Average income level [b]	Overall	/	0.095	0.583 (0.507-0.660)
	Low	Reference	Reference	/
	Moderate	0.442 (0.180-1.164)	0.083	/
	High	0.198 (0.028-0.902)	0.056	/
Vital Signs				
BMI		1.056 (0.936-1.191)	0.372	0.555 (0.457-0.654)
Right SBP		0.980 (0.956-1.003)	0.102	0.583 (0.490-0.676)
Right DBP		1.003 (0.970-1.036)	0.856	0.520 (0.423-0.617)
Left SBP		0.965 (0.940-0.989)	**0.007**	0.648 (0.557-0.739)
Left DBP		0.988 (0.953-1.022)	0.480	0.542 (0.443-0.642)
Average SBP		0.969 (0.943-0.995)	**0.023**	0.626 (0.537-0.716)
Average DBP		0.995 (0.959-1.031)	0.786	0.529 (0.435-0.622)
Past Medical History				
Current smoking		0.478 (0.137-1.296)	0.188	0.547 (0.489-0.605)
Current alcohol consumption		0.484 (0.111-1.465)	0.253	0.537 (0.485-0.588)
Diabetes		0.667 (0.270-1.490)	0.347	0.538 (0.463-0.613)
Hypertension		1.818 (0.792-4.721)	0.183	0.554 (0.482-0.626)

Dyslipidemia		2.550 (1.225-5.278)	**0.011**	0.605 (0.518-0.692)
All stroke		1.663 (0.817-3.398)	0.159	0.563 (0.474-0.651)
Ischemic stroke		1.228 (0.594-2.499)	0.574	0.525 (0.437-0.613)
ICH		16.500 (2.046-338.988)	**0.017**	0.538 (0.493-0.583)
TIA		0.447 (0.024-2.408)	0.447	0.516 (0.484-0.547)
Coronary heart disease		0.313 (0.049-1.109)	0.124	0.550 (0.505-0.595)
Atrial fibrillation		NA	0.990	0.511 (0.500-0.521)
Headache		0.944 (0.333-2.314)	0.905	0.504 (0.438-0.570)
Physical Activity				
Physical inactivity [c]		7.475 (1.580-39.415)	**0.011**	0.546 (0.495-0.598)
Average intensity [d]	Overall	/	0.077	0.522 (0.422-0.621)
	Vigorous	Reference	Reference	/
	Moderate	0.118 (0.022-0.578)	**0.008**	/
	Light	0.157 (0.028-0.784)	**0.024**	/
Vigorous-intensity physical activity		0.445 (0.160-1.057)	0.087	0.571 (0.502-0.639)
Moderate-intensity physical activity		0.565 (0.276-1.164)	0.118	0.567 (0.479-0.656)
Light-intensity physical activity		0.630 (0.307-1.277)	0.200	0.558 (0.469-0.646)
Functional Independence				
mRS	Overall	/	**0.019**	0.624 (0.535-0.712)
	0	Reference	Reference	/
	1	2.815 (1.349-5.949)	**0.006**	/
	2	2.667 (0.370-12.759)	0.253	/

**CSVD Neuroimaging Markers**				
RSSI				
Total RSSI count		0.967 (0.696-1.143)	0.767	0.517 (0.442-0.592)
Total RSSI volume (per 1,000 mm³)		1.072 (0.880-1.267)	0.398	0.521 (0.444-0.598)
Lacune				
Lacune count (based on FLAIR)		1.070 (0.982-1.161)	0.105	0.600 (0.497-0.704)
WMH				
Total WMH volume (per 1,000 mm³)		1.015 (0.998-1.032)	0.078	0.542 (0.433-0.651)
Total WMH volume percentage (% of total WM)		1.074 (0.992-1.159)	0.068	0.550 (0.441-0.659)
Periventricular rim WMH volume (per 1,000 mm³)		1.024 (0.997-1.050)	0.067	0.554 (0.445-0.663)
Periventricular rim WMH volume percentage (% of total WM)		1.114 (0.989-1.249)	0.065	0.558 (0.448-0.667)
Periventricular WMH volume (per 1,000 mm³)		1.061 (0.993-1.131)	0.071	0.564 (0.460-0.668)
Periventricular WMH volume percentage (% of total WM)		1.352 (0.983-1.845)	0.057	0.569 (0.466-0.673)
Deep WMH volume (per 1,000 mm³)		1.089 (0.956-1.229)	0.179	0.519 (0.406-0.633)
Deep WMH volume percentage (% of total WM)		1.564 (0.854-2.760)	0.130	0.522 (0.408-0.636)
Juxtacortical WMH volume (per 1,000 mm³)		0.892 (0.678-1.119)	0.368	0.563 (0.460-0.665)
Juxtacortical WMH volume percentage (% of total WM)		0.656 (0.189-1.825)	0.460	0.560 (0.457-0.664)
Fazekas score [e]	Overall	/	0.426	0.556 (0.461-0.651)
	1	Reference	Reference	/
	2	NA	0.992	/
	3	NA	0.992	/
	4	NA	0.992	/
	5	NA	0.992	/
	6	NA	1.000	/
Fazekas grade	Overall	/	0.527	0.548 (0.461-0.635)
	1	Reference	Reference	/
	2	1.336 (0.584-3.351)	0.511	/
	3	2.070 (0.560-7.139)	0.254	/

PVS			
Total PVS count (based on T2)	1.000 (0.997-1.003)	0.825	0.538 (0.442-0.635)
Total ePVS count (based on T2)	0.999 (0.993-1.005)	0.853	0.531 (0.436-0.626)
BG ePVS score (based on count >10)	2.421 (1.153-5.026)	0.018	0.597 (0.510-0.683)
CSO ePVS score (based on count >10)	1.010 (0.496-2.050)	0.978	0.501 (0.412-0.590)
Midbrain ePVS score (based on count >1)	0.580 (0.246-1.260)	0.187	0.557 (0.479-0.635)
Lateralized region-specific counts			
Left frontal lobe PVS count	1.001 (0.987-1.014)	0.884	0.516 (0.415-0.617)
Right frontal lobe PVS count	0.999 (0.984-1.013)	0.881	0.508 (0.408-0.608)
Left temporal lobe PVS count	0.987 (0.945-1.024)	0.522	0.506 (0.409-0.602)
Right temporal lobe PVS count	0.974 (0.927-1.013)	0.238	0.516 (0.424-0.608)
Left parietal lobe PVS count	1.002 (0.980-1.023)	0.841	0.534 (0.442-0.627)
Right parietal lobe PVS count	0.991 (0.968-1.012)	0.407	0.525 (0.431-0.620)
Left occipital lobe PVS count	0.883 (0.699-1.070)	0.245	0.539 (0.451-0.628)
Right occipital lobe PVS count	1.042 (0.894-1.196)	0.578	0.562 (0.465-0.659)
Left BG PVS count	1.030 (1.002-1.059)	**0.033**	0.621 (0.529-0.713)
Right BG PVS count	1.030 (1.001-1.061)	**0.044**	0.611 (0.511-0.712)
Left thalamus PVS count	1.128 (0.917-1.365)	0.229	0.531 (0.426-0.635)
Right thalamus PVS count	1.057 (0.861-1.262)	0.564	0.519 (0.418-0.619)
Left cerebellum PVS count	0.672 (0.040-2.502)	0.665	0.505 (0.476-0.535)
Right cerebellum PVS count	NA	0.989	0.505 (0.498-0.513)
Left CSO PVS count	1.011 (0.982-1.039)	0.459	0.545 (0.442-0.647)
Right CSO PVS count	0.994 (0.965-1.022)	0.698	0.505 (0.404-0.605)
Overall region-specific counts			
Overall frontal lobe PVS count	1.000 (0.993-1.007)	0.999	0.513 (0.412-0.613)

Overall temporal lobe PVS count	0.990 (0.966-1.009)	0.343	0.503 (0.411-0.595)
Overall parietal lobe PVS count	0.998 (0.987-1.009)	0.745	0.503 (0.408-0.597)
Overall occipital lobe PVS count	0.987 (0.892-1.080)	0.793	0.535 (0.442-0.627)
Overall BG PVS count	1.017 (1.002-1.032)	**0.029**	0.622 (0.526-0.717)
Overall thalamus PVS count	1.061 (0.942-1.182)	0.303	0.529 (0.421-0.638)
Overall cerebellum PVS count	0.552 (0.032-2.161)	0.539	0.510 (0.480-0.541)
Overall CSO PVS count	1.001 (0.986-1.015)	0.864	0.522 (0.420-0.624)
Overall midbrain PVS count	0.865 (0.558-1.228)	0.462	0.549 (0.471-0.626)
Overall pons PVS count	1.242 (0.958-1.592)	0.087	0.566 (0.474-0.659)
Overall medulla oblongata PVS count	0.718 (0.038-4.212)	0.760	0.505 (0.475-0.535)
Overall brainstem PVS count	1.084 (0.880-1.311)	0.423	0.538 (0.441-0.634)
Overall periventricular PVS count	NA	0.988	0.516 (0.503-0.529)
CMB			
Total CMB count	1.008 (0.981-1.031)	0.508	0.648 (0.552-0.743)
Total CMB volume (per 1,000 mm³)	0.933 (0.384-1.644)	0.841	0.645 (0.551-0.739)
Total CSVD burden [f]			
High TB-CSVD (vs. low)	3.135 (1.328-8.667)	**0.015**	0.608 (0.538-0.677)

Note: a: Absolute lesion volumes were rescaled per 1,000 mm³ in logistic regression models to improve interpretability of ORs. b: Average income level was defined as: Low (monthly household income per capita <2000 CNY), Moderate (monthly household income per capita 2000-<6000 CNY), and High (monthly household income per capita ≥6000 CNY). c: Physical inactivity was defined as no participation in any physical activity. d: Physical activity intensity was defined as: Light-intensity (1.5–<3 metabolic equivalent of tasks [METs]), Moderate-intensity (3–6 METs), and Vigorous-intensity (≥6 METs). e: The Fazekas score was divided into three categories: 1–2, 3–4, and 5–6. f: TB-CSVD was dichotomized as low burden (scores 0–1) and high burden (scores 2–4). ORs for high TB-CSVD indicate comparisons with the low-burden reference group. **Abbreviations:** BG, basal ganglia; CMB, cerebral microbleed; CSVD, cerebral small vessel disease; CSO, centrum semiovale; DBP, diastolic blood pressure; ePVS, enlarged perivascular spaces; FLAIR, fluid-attenuated inversion recovery; ICH, intracerebral hemorrhage; MCR, motoric cognitive risk syndrome; mRS, modified Rankin Scale; NA, not applicable due to zero cell count or model non-convergence; Extremely large ORs or confidence intervals containing 0/NA indicate sparse cells, quasi-complete separation, or model non-convergence and should be interpreted with caution; PVS, perivascular spaces; RSSI, recent subcortical small infarct; SBP, systolic blood pressure; TB-CSVD, the total cerebral small vessel disease burden score calculated according to Staals et al.; TIA, transient ischemic attack; WM, white matter; WMH, white matter hyperintensity.

Table S3. Univariable associations of demographic, clinical, and neuroimaging parameters with SCCs among patients with CSVD.

Variable	Level	OR (95% CI) [a]	*P*	AUC (95% CI)
**Demographic and Clinical Parameters**				
Demographics				
Age		0.947 (0.907-0.988)	**0.012**	0.393 (0.314-0.473)
Female		1.053 (0.583-1.928)	0.866	0.506 (0.436-0.576)
Years of education		0.896 (0.810-0.986)	**0.027**	0.413 (0.335-0.491)
Married		3.602 (1.202-11.374)	**0.023**	0.543 (0.500-0.586)
Solitary		1.583 (0.463-4.940)	0.436	0.513 (0.477-0.550)
Insured		0.667 (0.159-3.329)	0.587	0.493 (0.464-0.522)
Average income level [b]	Overall	/	0.766	0.478 (0.415-0.541)
	Low	Reference	Reference	/
	Moderate	0.879 (0.327-2.125)	0.783	/
	High	0.661 (0.197-2.145)	0.492	/
Vital Signs				
BMI		1.034 (0.936-1.144)	0.517	0.534 (0.450-0.617)
Right SBP		0.999 (0.980-1.018)	0.906	0.507 (0.423-0.591)
Right DBP		1.036 (1.006-1.068)	**0.021**	0.606 (0.524-0.688)
Left SBP		0.995 (0.977-1.014)	0.605	0.481 (0.398-0.564)
Left DBP		1.038 (1.008-1.070)	**0.015**	0.618 (0.535-0.702)
Average SBP		0.997 (0.978-1.016)	0.739	0.492 (0.408-0.576)
Average DBP		1.041 (1.010-1.076)	**0.012**	0.617 (0.534-0.699)
Past Medical History				
Current smoking		0.885 (0.434-1.883)	0.744	0.491 (0.433-0.548)

Current alcohol consumption		0.396 (0.184-0.859)	**0.018**	0.438 (0.381-0.495)
Diabetes		0.335 (0.180-0.621)	**0.001**	0.383 (0.314-0.452)
Hypertension		1.089 (0.568-2.042)	0.793	0.509 (0.443-0.574)
Dyslipidemia		1.312 (0.690-2.589)	0.418	0.527 (0.463-0.591)
All stroke		1.385 (0.767-2.542)	0.285	0.539 (0.469-0.609)
Ischemic stroke		1.372 (0.757-2.537)	0.303	0.537 (0.468-0.606)
ICH		1.223 (0.153-24.974)	0.863	0.502 (0.483-0.520)
TIA		1.232 (0.354-5.690)	0.760	0.505 (0.474-0.536)
Coronary heart disease		0.831 (0.375-1.947)	0.656	0.489 (0.437-0.540)
Atrial fibrillation		1.223 (0.153-24.974)	0.863	0.502 (0.483-0.520)
Headache		3.118 (1.256-9.459)	**0.024**	0.565 (0.519-0.610)
Physical Activity				
Physical inactivity [c]		0.401 (0.021-2.412)	0.402	0.489 (0.468-0.510)
Average intensity [d]	Overall	/	0.066	0.402 (0.329-0.474)
	Vigorous	Reference	Reference	/
	Moderate	0.543 (0.028-3.348)	0.579	/
	Light	0.255 (0.013-1.591)	0.216	/
Vigorous-intensity physical activity		1.139 (0.602-2.225)	0.694	0.513 (0.449-0.577)
Moderate-intensity physical activity		1.262 (0.689-2.288)	0.446	0.527 (0.457-0.597)
Light-intensity physical activity		0.708 (0.390-1.269)	0.251	0.458 (0.387-0.529)
Functional Independence				
mRS	Overall	/	0.402	0.533 (0.463-0.602)
	0	Reference	Reference	/
	1	1.497 (0.794-2.916)	0.221	/
	2	0.758 (0.178-3.821)	0.712	/

**CSVD Neuroimaging Markers**				
RSSI				
Total RSSI count		0.891 (0.726-1.032)	0.176	0.476 (0.415-0.537)
Total RSSI volume (per 1,000 mm³)		0.867 (0.721-1.042)	0.128	0.468 (0.406-0.531)
Lacune				
Lacune count (based on FLAIR)		1.049 (0.967-1.155)	0.285	0.566 (0.488-0.643)
WMH				
Total WMH volume (per 1,000 mm³)		1.008 (0.992-1.024)	0.344	0.568 (0.481-0.654)
Total WMH volume percentage (% of total WM)		1.030 (0.959-1.114)	0.438	0.564 (0.478-0.650)
Periventricular rim WMH volume (per 1,000 mm³)		1.008 (0.984-1.033)	0.515	0.563 (0.476-0.649)
Periventricular rim WMH volume percentage (% of total WM)		1.028 (0.924-1.157)	0.629	0.561 (0.475-0.648)
Periventricular WMH volume (per 1,000 mm³)		1.016 (0.956-1.079)	0.614	0.529 (0.445-0.613)
Periventricular WMH volume percentage (% of total WM)		1.046 (0.791-1.410)	0.759	0.523 (0.439-0.607)
Deep WMH volume (per 1,000 mm³)		1.074 (0.950-1.214)	0.253	0.550 (0.466-0.634)
Deep WMH volume percentage (% of total WM)		1.373 (0.794-2.537)	0.280	0.547 (0.462-0.631)
Juxtacortical WMH volume (per 1,000 mm³)		1.420 (1.105-1.826)	**0.006**	0.610 (0.532-0.688)
Juxtacortical WMH volume percentage (% of total WM)		4.968 (1.704-17.582)	**0.007**	0.606 (0.528-0.684)
Fazekas score [e]	Overall	/	**0.022**	0.584 (0.507-0.662)
	1	Reference	Reference	/
	2	11.176 (1.641-222.989)	**0.033**	/
	3	12.308 (1.765-248.290)	**0.028**	/
	4	12.500 (1.906-245.260)	**0.024**	/
	5	40.000 (4.059-1042.111)	**0.005**	/
	6	2.879×10^7 (0.000-NA)	0.984	/
Fazekas grade	Overall	/	**0.045**	0.575 (0.507-0.643)

	1	Reference	Reference	/
	2	1.403 (0.737-2.642)	0.296	/
	3	5.359 (1.377-35.632)	**0.034**	/
PVS				
Total PVS count (based on T2)		0.998 (0.996-1.001)	0.209	0.442 (0.360-0.524)
Total ePVS count (based on T2)		0.996 (0.991-1.000)	0.076	0.410 (0.331-0.490)
BG ePVS score (based on count >10)		1.345 (0.699-2.695)	0.387	0.528 (0.466-0.591)
CSO ePVS score (based on count >10)		0.804 (0.450-1.433)	0.460	0.473 (0.400-0.545)
Midbrain ePVS score (based on count >1)		0.996 (0.545-1.851)	0.989	0.500 (0.431-0.568)
Lateralized region-specific counts				
Left frontal lobe PVS count		0.991 (0.980-1.002)	0.099	0.421 (0.341-0.502)
Right frontal lobe PVS count		0.990 (0.979-1.001)	0.076	0.414 (0.333-0.494)
Left temporal lobe PVS count		0.987 (0.959-1.016)	0.359	0.466 (0.382-0.550)
Right temporal lobe PVS count		0.985 (0.958-1.014)	0.302	0.438 (0.357-0.520)
Left parietal lobe PVS count		0.986 (0.969-1.003)	0.113	0.413 (0.333-0.493)
Right parietal lobe PVS count		0.985 (0.968-1.002)	0.078	0.419 (0.337-0.501)
Left occipital lobe PVS count		0.926 (0.805-1.069)	0.282	0.471 (0.393-0.549)
Right occipital lobe PVS count		0.974 (0.865-1.104)	0.673	0.444 (0.367-0.521)
Left BG PVS count		1.017 (0.992-1.043)	0.199	0.546 (0.463-0.629)
Right BG PVS count		1.010 (0.985-1.036)	0.443	0.530 (0.447-0.613)
Left thalamus PVS count		0.989 (0.832-1.189)	0.901	0.473 (0.396-0.550)
Right thalamus PVS count		1.033 (0.878-1.242)	0.707	0.510 (0.430-0.591)
Left cerebellum PVS count		1.389 (0.457-8.238)	0.625	0.503 (0.478-0.529)
Right cerebellum PVS count		2.369×10^{6} (0.000-NA)	0.989	0.506 (0.498-0.515)
Left CSO PVS count		0.993 (0.970-1.016)	0.533	0.461 (0.381-0.541)
Right CSO PVS count		0.991 (0.969-1.014)	0.424	0.454 (0.373-0.535)
Overall region-specific counts				
Overall frontal lobe PVS count		0.995 (0.989-1.001)	0.081	0.417 (0.336-0.497)

Overall temporal lobe PVS count	0.992 (0.978-1.008)	0.311	0.451 (0.368-0.535)
Overall parietal lobe PVS count	0.992 (0.983-1.001)	0.085	0.415 (0.334-0.496)
Overall occipital lobe PVS count	0.970 (0.903-1.046)	0.417	0.442 (0.364-0.521)
Overall BG PVS count	1.007 (0.994-1.021)	0.279	0.546 (0.463-0.629)
Overall thalamus PVS count	1.008 (0.914-1.121)	0.875	0.499 (0.417-0.581)
Overall cerebellum PVS count	1.703 (0.552-10.534)	0.451	0.510 (0.483-0.537)
Overall CSO PVS count	0.996 (0.984-1.008)	0.460	0.456 (0.376-0.536)
Overall midbrain PVS count	1.055 (0.800-1.435)	0.717	0.518 (0.451-0.585)
Overall pons PVS count	1.098 (0.866-1.439)	0.466	0.506 (0.434-0.578)
Overall medulla oblongata PVS count	0.667 (0.159-3.329)	0.587	0.493 (0.464-0.522)
Overall brainstem PVS count	1.066 (0.897-1.286)	0.487	0.515 (0.436-0.594)
Overall periventricular PVS count	1.131 (0.541-3.377)	0.770	0.508 (0.487-0.528)
CMB			
Total CMB count	1.004 (0.982-1.031)	0.754	0.582 (0.503-0.661)
Total CMB volume (per 1,000 mm³)	1.316 (0.686-2.526)	0.409	0.576 (0.496-0.655)
Total CSVD burden [f]			
High TB-CSVD (vs. low)	2.478 (1.367-4.516)	**0.003**	0.606 (0.535-0.676)

Note: a: Absolute lesion volumes were rescaled per 1,000 mm³ in logistic regression models to improve interpretability of ORs. b: Average income level was defined as: Low (monthly household income per capita <2000 CNY), Moderate (monthly household income per capita 2000-<6000 CNY), and High (monthly household income per capita ≥6000 CNY). c: Physical inactivity was defined as no participation in any physical activity. d: Physical activity intensity was defined as: Light-intensity (1.5–<3 metabolic equivalent of tasks [METs]), Moderate-intensity (3–6 METs), and Vigorous-intensity (≥6 METs). e: The Fazekas score was divided into three categories: 1–2, 3–4, and 5–6. f: TB-CSVD was dichotomized as low burden (scores 0–1) and high burden (scores 2–4). ORs for high TB-CSVD indicate comparisons with the low-burden reference group. **Abbreviations:** BG, basal ganglia; CMB, cerebral microbleed; CSVD, cerebral small vessel disease; CSO, centrum semiovale; DBP, diastolic blood pressure; ePVS, enlarged perivascular spaces; FLAIR, fluid-attenuated inversion recovery; ICH, intracerebral hemorrhage; MCR, motoric cognitive risk syndrome; mRS, modified Rankin Scale; NA, not applicable due to zero cell count or model non-convergence; Extremely large ORs or confidence intervals containing 0/NA indicate sparse cells, quasi-complete separation, or model non-convergence and

should be interpreted with caution; PVS, perivascular spaces; RSSI, recent subcortical small infarct; SBP, systolic blood pressure; TB-CSVD, the total cerebral small vessel disease burden score calculated according to Staals et al.; TIA, transient ischemic attack; WM, white matter; WMH, white matter hyperintensity.

Table S4. Univariable associations of demographic, clinical, and neuroimaging parameters with slow gait among patients with CSVD.

Variable	Level	OR (95% CI) [a]	*P*	AUC (95% CI)
**Demographic and Clinical Parameters**				
Demographics				
Age		0.983 (0.939-1.028)	0.454	0.531 (0.444-0.619)
Female		1.528 (0.821-2.832)	0.178	0.551 (0.475-0.626)
Years of education		0.951 (0.860-1.051)	0.328	0.465 (0.382-0.548)
Married		1.199 (0.358-5.450)	0.787	0.505 (0.470-0.541)
Solitary		1.083 (0.317-4.966)	0.906	0.502 (0.467-0.537)
Insured		0.431 (0.023-2.500)	0.436	0.489 (0.465-0.512)
Average income level [b]	Overall	/	**0.044**	0.418 (0.353-0.482)
	Low	Reference	Reference	/
	Moderate	0.455 (0.197-1.081)	0.068	/
	High	0.190 (0.038-0.720)	**0.022**	/
Vital Signs				
BMI		1.044 (0.941-1.159)	0.416	0.538 (0.452-0.624)
Right SBP		0.976 (0.955-0.997)	**0.026**	0.604 (0.524-0.684)
Right DBP		0.983 (0.953-1.012)	0.251	0.580 (0.497-0.662)
Left SBP		0.967 (0.945-0.987)	**0.003**	0.643 (0.564-0.721)
Left DBP		0.969 (0.939-0.999)	**0.047**	0.599 (0.515-0.683)
Average SBP		0.968 (0.945-0.990)	**0.006**	0.632 (0.554-0.710)
Average DBP		0.973 (0.941-1.004)	0.096	0.592 (0.510-0.674)
Past Medical History				
Current smoking		0.359 (0.119-0.891)	**0.043**	0.437 (0.387-0.486)
Current alcohol consumption		0.398 (0.114-1.076)	0.099	0.454 (0.410-0.499)
Diabetes		1.074 (0.538-2.077)	0.836	0.507 (0.438-0.577)

Hypertension		1.186 (0.604-2.430)	0.629	0.517 (0.449-0.584)
Dyslipidemia		2.032 (1.062-3.856)	**0.031**	0.576 (0.503-0.649)
All stroke		1.281 (0.691-2.365)	0.429	0.530 (0.454-0.606)
Ischemic stroke		0.950 (0.503-1.764)	0.871	0.494 (0.419-0.568)
ICH		1.919×10^{7} (0.000-NA)	0.982	0.536 (0.502-0.571)
TIA		0.268 (0.014-1.425)	0.212	0.477 (0.451-0.502)
Coronary heart disease		0.415 (0.119-1.127)	0.117	0.457 (0.413-0.501)
Atrial fibrillation		NA	0.984	0.488 (0.477-0.500)
Headache		0.654 (0.251-1.506)	0.346	0.472 (0.419-0.526)
Physical Activity				
Physical inactivity [c]		0.229 (0.044-1.071)	0.059	0.472 (0.436-0.508)
Average intensity [d]	Overall	/	0.060	0.524 (0.440-0.608)
	Vigorous	Reference	Reference	/
	Moderate	0.175 (0.033-0.842)	**0.029**	/
	Light	0.316 (0.058-1.535)	0.150	/
Vigorous-intensity physical activity		0.356 (0.147-0.768)	**0.013**	0.411 (0.352-0.470)
Moderate-intensity physical activity		0.402 (0.214-0.749)	**0.004**	0.607 (0.532-0.682)
Light-intensity physical activity		0.780 (0.423-1.439)	0.426	0.469 (0.393-0.546)
Functional Independence				
mRS	Overall	/	**0.009**	0.616 (0.539-0.692)
	0	Reference	Reference	/
	1	2.364 (1.241-4.515)	**0.009**	/
	2	4.538 (1.015-20.353)	**0.041**	/
**CSVD Neuroimaging Markers**				
RSSI				
Total RSSI count		1.003 (0.816-1.160)	0.975	0.515 (0.451-0.579)

Total RSSI volume (per 1,000 mm³)		1.056 (0.903-1.234)	0.494	0.522 (0.456-0.588)
Lacune				
Lacune count (based on FLAIR)		1.036 (0.955-1.118)	0.373	0.556 (0.469-0.643)
WMH				
Total WMH volume (per 1,000 mm³)		1.004 (0.988-1.020)	0.640	0.551 (0.456-0.646)
Total WMH volume percentage (% of total WM)		1.027 (0.954-1.102)	0.463	0.537 (0.442-0.633)
Periventricular rim WMH volume (per 1,000 mm³)		1.009 (0.986-1.034)	0.436	0.538 (0.442-0.633)
Periventricular rim WMH volume percentage (% of total WM)		1.055 (0.943-1.172)	0.331	0.530 (0.434-0.627)
Periventricular WMH volume (per 1,000 mm³)		1.019 (0.960-1.082)	0.532	0.493 (0.402-0.583)
Periventricular WMH volume percentage (% of total WM)		1.152 (0.860-1.529)	0.329	0.493 (0.402-0.585)
Deep WMH volume (per 1,000 mm³)		1.002 (0.890-1.128)	0.971	0.556 (0.462-0.650)
Deep WMH volume percentage (% of total WM)		1.078 (0.605-1.837)	0.789	0.551 (0.456-0.645)
Juxtacortical WMH volume (per 1,000 mm³)		0.737 (0.569-0.954)	**0.021**	0.638 (0.554-0.723)
Juxtacortical WMH volume percentage (% of total WM)		0.287 (0.081-0.828)	**0.035**	0.631 (0.545-0.717)
Fazekas score [e]	Overall	/	0.567	0.530 (0.443-0.616)
	1	Reference	Reference	/
	2	0.821 (0.145-6.341)	0.829	/
	3	0.571 (0.096-4.549)	0.550	/
	4	0.545 (0.099-4.126)	0.501	/
	5	1.000 (0.146-8.723)	1.000	/
	6	NA	0.986	/
Fazekas grade	Overall	/	0.448	0.474 (0.395-0.553)
	1	Reference	Reference	/
	2	0.661 (0.337-1.319)	0.232	/
	3	0.956 (0.301-2.774)	0.935	/
PVS				
Total PVS count (based on T2)		1.002 (0.999-1.004)	0.218	0.578 (0.495-0.661)

Total ePVS count (based on T2)	1.003 (0.998-1.008)	0.245	0.589 (0.508-0.670)
BG ePVS score (based on count >10)	2.239 (1.166-4.273)	**0.015**	0.585 (0.513-0.658)
CSO ePVS score (based on count >10)	1.255 (0.682-2.317)	0.465	0.528 (0.452-0.605)
Midbrain ePVS score (based on count >1)	0.939 (0.485-1.775)	0.850	0.493 (0.421-0.565)
Lateralized region-specific counts			
Left frontal lobe PVS count	1.007 (0.996-1.018)	0.214	0.563 (0.477-0.650)
Right frontal lobe PVS count	1.006 (0.994-1.018)	0.303	0.561 (0.476-0.646)
Left temporal lobe PVS count	1.000 (0.968-1.031)	0.985	0.539 (0.453-0.626)
Right temporal lobe PVS count	0.989 (0.954-1.021)	0.513	0.526 (0.445-0.606)
Left parietal lobe PVS count	1.015 (0.997-1.033)	0.103	0.598 (0.519-0.678)
Right parietal lobe PVS count	1.011 (0.993-1.029)	0.240	0.574 (0.491-0.657)
Left occipital lobe PVS count	0.955 (0.804-1.114)	0.578	0.464 (0.385-0.543)
Right occipital lobe PVS count	1.025 (0.898-1.161)	0.702	0.550 (0.467-0.633)
Left BG PVS count	1.019 (0.995-1.045)	0.124	0.578 (0.495-0.661)
Right BG PVS count	1.018 (0.992-1.044)	0.177	0.569 (0.483-0.655)
Left thalamus PVS count	1.109 (0.924-1.321)	0.252	0.549 (0.464-0.633)
Right thalamus PVS count	1.047 (0.877-1.231)	0.591	0.521 (0.436-0.607)
Left cerebellum PVS count	0.451 (0.025-1.811)	0.418	0.488 (0.465-0.512)
Right cerebellum PVS count	NA	0.989	0.494 (0.486-0.502)
Left CSO PVS count	1.023 (0.998-1.048)	0.066	0.598 (0.514-0.682)
Right CSO PVS count	1.012 (0.988-1.035)	0.318	0.570 (0.485-0.654)
Overall region-specific counts			
Overall frontal lobe PVS count	1.003 (0.998-1.009)	0.246	0.563 (0.477-0.648)
Overall temporal lobe PVS count	0.997 (0.979-1.013)	0.740	0.540 (0.457-0.623)
Overall parietal lobe PVS count	1.007 (0.997-1.016)	0.149	0.587 (0.506-0.669)
Overall occipital lobe PVS count	0.998 (0.918-1.078)	0.958	0.527 (0.444-0.610)
Overall BG PVS count	1.010 (0.997-1.024)	0.129	0.574 (0.489-0.658)
Overall thalamus PVS count	1.051 (0.947-1.160)	0.331	0.548 (0.460-0.635)

Overall cerebellum PVS count	0.354 (0.020-1.516)	0.308	0.483 (0.458-0.507)
Overall CSO PVS count	1.009 (0.997-1.022)	0.144	0.586 (0.502-0.670)
Overall midbrain PVS count	0.976 (0.706-1.299)	0.872	0.499 (0.429-0.570)
Overall pons PVS count	1.247 (0.989-1.575)	0.059	0.576 (0.497-0.656)
Overall medulla oblongata PVS count	0.431 (0.023-2.500)	0.436	0.489 (0.465-0.512)
Overall brainstem PVS count	1.114 (0.934-1.320)	0.218	0.561 (0.478-0.643)
Overall periventricular PVS count	NA	0.987	0.482 (0.468-0.496)
CMB			
Total CMB count	1.002 (0.977-1.024)	0.849	0.554 (0.465-0.642)
Total CMB volume (per 1,000 mm^3)	0.815 (0.423-1.570)	0.541	0.554 (0.466-0.642)
Total CSVD burden [f]			
High TB-CSVD (vs. low)	1.093 (0.579-2.117)	0.788	0.510 (0.438-0.582)

Note: a: Absolute lesion volumes were rescaled per 1,000 mm^3 in logistic regression models to improve interpretability of ORs. b: Average income level was defined as: Low (monthly household income per capita <2000 CNY), Moderate (monthly household income per capita 2000-<6000 CNY), and High (monthly household income per capita ≥6000 CNY). c: Physical inactivity was defined as no participation in any physical activity. d: Physical activity intensity was defined as: Light-intensity (1.5–<3 metabolic equivalent of tasks [METs]), Moderate-intensity (3–6 METs), and Vigorous-intensity (≥6 METs). e: The Fazekas score was divided into three categories: 1–2, 3–4, and 5–6. f: TB-CSVD was dichotomized as low burden (scores 0–1) and high burden (scores 2–4). ORs for high TB-CSVD indicate comparisons with the low-burden reference group. **Abbreviations:** BG, basal ganglia; CMB, cerebral microbleed; CSVD, cerebral small vessel disease; CSO, centrum semiovale; DBP, diastolic blood pressure; ePVS, enlarged perivascular spaces; FLAIR, fluid-attenuated inversion recovery; ICH, intracerebral hemorrhage; MCR, motoric cognitive risk syndrome; mRS, modified Rankin Scale; NA, not applicable due to zero cell count or model non-convergence; Extremely large ORs or confidence intervals containing 0/NA indicate sparse cells, quasi-complete separation, or model non-convergence and should be interpreted with caution; PVS, perivascular spaces; RSSI, recent subcortical small infarct; SBP, systolic blood pressure; TB-CSVD, the total cerebral small vessel disease burden score calculated according to Staals et al.; TIA, transient ischemic attack; WM, white matter; WMH, white matter hyperintensity.

Table S5. Firth penalized logistic regression estimates for exploratory multivariable models and physical-activity sensitivity analyses.

Outcome	Model	Variable	Firth OR (95% CI)	*P*
MCR	Model 1: Ref. + Average SBP	Average SBP	0.968 (0.941-0.994)	**0.016**
	Model 2: Ref. + Physical inactivity	Physical inactivity	6.681 (1.516-32.025)	**0.013**
	Model 3: Ref. + High TB-CSVD	High TB-CSVD	2.968 (1.263-8.013)	**0.011**
	Model 4: Ref. + Average SBP + Physical inactivity + High TB-CSVD	Average SBP	0.967 (0.939-0.993)	**0.013**
		Physical inactivity	6.888 (1.408-38.084)	**0.018**
		High TB-CSVD	3.143 (1.306-8.680)	**0.009**
	Sensitivity: Ref. + Average SBP + No MVPA + High TB-CSVD	Average SBP	0.969 (0.942-0.995)	**0.020**
		No moderate-to-vigorous physical activity	1.846 (0.854-3.966)	0.118
		High TB-CSVD	3.099 (1.297-8.494)	**0.010**
	Sensitivity: Ref. + Average SBP + MVPA + High TB-CSVD	Average SBP	0.969 (0.942-0.995)	**0.020**
		Moderate-to-vigorous physical activity	0.542 (0.252-1.171)	0.118
		High TB-CSVD	3.099 (1.297-8.494)	**0.010**
	Sensitivity: Ref. + Average SBP + Physical-activity intensity + High TB-CSVD	Average SBP	0.968 (0.940-0.995)	**0.018**
		Physical-activity intensity: moderate	0.695 (0.314-1.555)	0.372
		Physical-activity intensity: vigorous	1.754 (0.150-13.002)	0.610
		Physical-activity intensity: none	5.537 (1.066-32.626)	**0.042**
		High TB-CSVD	3.227 (1.318-9.098)	**0.009**
SCCs	Model 1: Ref. + Average DBP + Headache	Average DBP	1.032 (0.999-1.067)	0.056
		Headache	2.580 (1.033-7.627)	**0.042**
		Juxtacortical WMH volume (per 1,000 mm^3)	1.392 (1.094-1.849)	**0.005**

Outcome	Model	Variable	Firth OR (95% CI)	*P*
	Model 2: Ref. + Juxtacortical WMH volume (per 1,000 mm³) + High TB-CSVD	High TB-CSVD	2.000 (1.064-3.765)	**0.031**
	Model 3: Ref. + Average DBP + Headache + Juxtacortical WMH volume (per 1,000 mm³) + High TB-CSVD	Average DBP	1.028 (0.994-1.064)	0.105
		Headache	2.389 (0.937-7.152)	0.069
		Juxtacortical WMH volume (per 1,000 mm³)	1.381 (1.084-1.839)	**0.007**
		High TB-CSVD	1.842 (0.965-3.514)	0.064
Slow gait	Model 1: Ref. + Average SBP + Dyslipidemia + mRS	Average SBP	0.964 (0.940-0.987)	**0.002**
		Dyslipidemia	2.053 (1.047-4.014)	**0.036**
		mRS	2.481 (1.444-4.362)	**<0.001**
	Model 2: Ref. + Moderate-intensity physical activity	Moderate-intensity physical activity	0.392 (0.209-0.731)	**0.003**
	Model 3: Ref. + Juxtacortical WMH volume (per 1,000 mm³)	Juxtacortical WMH volume (per 1,000 mm³)	0.758 (0.574-0.955)	**0.016**
	Model 4: Ref. + Average SBP + Dyslipidemia + mRS + Moderate-intensity physical activity + Juxtacortical WMH volume (per 1,000 mm³)	Average SBP	0.965 (0.940-0.988)	**0.003**
		Dyslipidemia	2.079 (1.037-4.163)	**0.039**
		mRS	2.348 (1.345-4.171)	**0.003**
		Moderate-intensity physical activity	0.530 (0.271-1.037)	0.064
		Juxtacortical WMH volume (per 1,000 mm³)	0.774 (0.580-0.986)	**0.037**

Note: Firth penalized logistic regression was used to reduce small-sample and sparse-data bias. ORs are coded as odds of the positive outcome (MCR, SCCs, or slow gait). Ref. includes age, sex, and years of education. TB-CSVD was dichotomized as low burden (scores 0–1) and high burden (scores 2–4). ORs for high TB-CSVD indicate comparisons with the low-burden reference group. Absolute lesion volumes were rescaled per 1,000 mm³ in

Firth penalized logistic regression models to improve interpretability of ORs. **Abbreviations:** CSVD, cerebral small vessel disease; DBP, diastolic blood pressure; MCR, motoric cognitive risk syndrome; mRS, modified Rankin Scale; MVPA, moderate-to-vigorous physical activity; OR, odds ratio; SBP, systolic blood pressure; SCCs, subjective cognitive complaints; TB-CSVD, the total cerebral small vessel disease burden score calculated according to Staals et al.; WMH, white matter hyperintensity.

Table S6. Bootstrap internal validation and optimism-corrected AUCs for exploratory multivariable models.

Outcome	Model	N	Events	Parameters	EPV	Apparent AUC	Optimism-corrected AUC
MCR	Reference: demographics	225	37	3	12.33	0.636	0.597
	Model 1: Ref. + Average SBP	225	37	4	9.25	0.697	0.657
	Model 2: Ref. + Physical inactivity	225	37	4	9.25	0.666	0.628
	Model 3: Ref. + High TB-CSVD	225	37	4	9.25	0.684	0.645
	Model 4: Ref. + Average SBP + Physical inactivity + High TB-CSVD	225	37	6	6.17	0.732	0.691
	Sensitivity: Ref. + Average SBP + No MVPA + High TB-CSVD	225	37	6	6.17	0.740	0.694
	Sensitivity: Ref. + Average SBP + MVPA + High TB-CSVD	225	37	6	6.17	0.740	0.696
	Sensitivity: Ref. + Average SBP + Physical-activity intensity + High TB-CSVD	225	37	8	4.62	0.741	0.684
SCCs	Model 1: Ref. + Average DBP + Headache	225	160	5	32.00	0.687	0.658
	Model 2: Ref. + Juxtacortical WMH volume (per 1,000 mm^3) + High TB-CSVD	225	160	5	32.00	0.722	0.690
	Model 3: Ref. + Average DBP + Headache + Juxtacortical WMH volume (per 1,000 mm^3) + High TB-CSVD	225	160	7	22.86	0.733	0.696
Slow gait	Model 1: Ref. + Average SBP + Dyslipidemia + mRS	225	55	6	9.17	0.721	0.685
	Model 2: Ref. + Moderate-intensity physical activity	225	55	4	13.75	0.646	0.611
	Model 3: Ref. + Juxtacortical WMH volume (per 1,000 mm^3)	225	55	4	13.75	0.664	0.624

Outcome	Model	N	Events	Parameters	EPV	Apparent AUC	Optimism-corrected AUC
	Model 4: Ref. + Average SBP + Dyslipidemia + mRS + Moderate-intensity physical activity + Juxtacortical WMH volume (per 1,000 mm^3)	225	55	8	6.88	0.770	0.725

Note: Optimism-corrected AUCs are presented as point estimates from 1,000 bootstrap resamples. EPV was calculated as the number of outcome events divided by the number of predictor parameters excluding the intercept. Ref. includes age, sex, and years of education. TB-CSVD was dichotomized as low burden (scores 0–1) and high burden (scores 2–4). **Abbreviations:** AUC, area under the receiver operating characteristic curve; CSVD, cerebral small vessel disease; DBP, diastolic blood pressure; EPV, events per variable; MCR, motoric cognitive risk syndrome; mRS, modified Rankin Scale; MVPA, moderate-to-vigorous physical activity; SBP, systolic blood pressure; SCCs, subjective cognitive complaints; TB-CSVD, the total cerebral small vessel disease burden score calculated according to Staals et al.; WMH, white matter hyperintensity.

Table S7. Sparse-exposure distributions for key binary variables used in multivariable and sensitivity analyses.

Outcome	Exposure	Outcome level	Exposure = 0	Exposure = 1
MCR	Physical inactivity	0	185	3
		1	33	4
	No MVPA	0	131	57
		1	21	16
	High TB-CSVD	0	71	117
		1	6	31
SCCs	High TB-CSVD	0	32	33
		1	45	115
Slow gait	Moderate-intensity physical activity	0	50	120
		1	28	27

Note: Outcome level 0 indicates absence of the outcome and outcome level 1 indicates presence of the outcome. Exposure = 0 and Exposure = 1 indicate absence and presence of the exposure, respectively. For MCR, physical inactivity was present in 7/225 participants, including 4/37 participants with MCR and 3/188 without MCR. This sparse distribution supports cautious interpretation of physical-activity estimates. TB-CSVD was dichotomized as low burden (scores 0–1) and high burden (scores 2–4). ORs for high TB-CSVD indicate comparisons with the low-burden reference group. **Abbreviations:** MCR, motoric cognitive risk syndrome; MVPA, moderate-to-vigorous physical activity; SCCs, subjective cognitive complaints; TB-CSVD, the total cerebral small vessel disease burden score calculated according to Staals et al.

Table S8. Pairwise DeLong comparisons of apparent AUCs between exploratory multivariable models.

Outcome	Model A	Model B	AUC of Model A	AUC of Model B	Difference in AUC (Model B - Model A)	DeLong *P*	N	Events
MCR	Reference	Model 1	0.636	0.697	0.062	0.081	225	37
	Reference	Model 2	0.636	0.666	0.031	0.219	225	37
	Reference	Model 3	0.636	0.684	0.048	0.214	225	37
	Reference	Model 4	0.636	0.732	0.097	**0.036**	225	37
	Model 1	Model 4	0.697	0.732	0.035	0.321	225	37
	Model 2	Model 4	0.666	0.732	0.066	0.098	225	37
	Model 3	Model 4	0.684	0.732	0.049	0.115	225	37
SCCs	Reference	Model 1	0.654	0.687	0.033	0.239	225	160
	Reference	Model 2	0.654	0.722	0.068	0.058	225	160
	Reference	Model 3	0.654	0.733	0.079	**0.032**	225	160
	Model 1	Model 3	0.687	0.733	0.047	0.092	225	160
	Model 2	Model 3	0.722	0.733	0.011	0.564	225	160
Slow gait	Reference	Model 1	0.575	0.721	0.146	**0.003**	225	55
	Reference	Model 2	0.575	0.646	0.071	0.080	225	55
	Reference	Model 3	0.575	0.664	0.089	**0.008**	225	55
	Reference	Model 4	0.575	0.770	0.195	**<0.001**	225	55
	Model 1	Model 4	0.721	0.770	0.049	**0.033**	225	55
	Model 2	Model 4	0.646	0.770	0.124	**0.001**	225	55
	Model 3	Model 4	0.664	0.770	0.106	**0.017**	225	55

Note: Pairwise DeLong tests were used to compare apparent AUCs between models fitted on the same complete-case dataset for each outcome. Difference in AUC was calculated as model B minus model A. DeLong tests were performed for apparent AUCs from the original dataset; optimism-corrected AUCs were not compared using DeLong tests. Reference includes age, sex, and years of education. **Model definitions:** For MCR, Model 1 = Reference + average SBP; Model 2 = Reference + physical inactivity; Model 3 = Reference + high TB-CSVD; Model 4 = Reference + average SBP + physical inactivity + high TB-CSVD. For SCCs, Model 1 = Reference + average DBP + headache; Model 2 = Reference + juxtacortical WMH volume (per 1,000 mm^3) + high TB-CSVD; Model 3 = Reference + average DBP + headache + juxtacortical WMH volume (per 1,000 mm^3) + high TB-CSVD. For slow gait, Model 1 = Reference + average SBP + dyslipidemia + mRS; Model 2 = Reference + moderate-intensity physical activity; Model 3 = Reference + juxtacortical WMH volume (per 1,000 mm^3); Model 4 = Reference + average SBP + dyslipidemia + mRS + moderate-intensity physical activity + juxtacortical WMH volume (per 1,000 mm^3). **Interpretation:** For MCR, Model 4 significantly outperformed the demographic reference model but did not significantly outperform the single-domain models. For SCCs, Model 3 significantly outperformed the demographic reference model but not the single-domain models. For slow gait, Model 4 significantly outperformed both the demographic reference model and the single-domain models. **Abbreviations:** AUC, area under the receiver operating characteristic curve; CSVD, cerebral small vessel disease; DBP, diastolic blood pressure; MCR, motoric cognitive risk syndrome; mRS, modified Rankin Scale; SBP, systolic blood pressure; SCCs, subjective cognitive complaints; TB-CSVD, the total cerebral small vessel disease burden score calculated according to Staals et al.; WMH, white matter hyperintensity.
